# A Family of Early English Oculists (1600-1751), With a Reappraisal of John Thomas Woolhouse (1664-1733/1734)

**DOI:** 10.1177/1179172117732042

**Published:** 2017-09-29

**Authors:** Christopher T Leffler, Stephen G Schwartz

**Affiliations:** 1 Department of Ophthalmology, Virginia Commonwealth University, Richmond, VA, USA; 2 Bascom Palmer Eye Institute, University of Miami, Miller School of Medicine, Naples, FL, USA

**Keywords:** Medical history, cataract couching, glaucoma

## Abstract

**Introduction:**

John Thomas Woolhouse (1666-1733/1734), who practiced in Paris, was part of a family with 5 generations of English oculists. Some historians have derided him as a “charlatan” and have criticized him for adhering to the old notion that a cataract was a membrane anterior to the lens.

**Methods:**

We reviewed treatises and digital records related to Woolhouse and his family and the handwritten notes of his 1721 lecture series at the Royal Society of Medicine.

**Results:**

We have identified 5 generations of oculists in Woolhouse’s family, by the names of Atwood, Stepkins, Ivy, and Beaumont. Woolhouse taught students from across Europe. He was one of the early proponents in Europe, inspired by Asian medical practices, to perform paracentesis to release aqueous for a new condition called hydrophthalmia. In Woolhouse’s system, some of these cases probably described angle-closure glaucoma. He was the first to attach the name glaucoma to the palpably hard eye in 1707. He may also have been the first to teach that a soft eye was unlikely to recover vision. Credit for these teachings has traditionally gone to one of his students, Johannes Zacharias Platner, in 1745. Some historians have stated that he proposed iridectomy as a theoretical procedure, which was later performed by Cheselden. In fact, Woolhouse described techniques he had performed which today would be called pupilloplasty, synechiolysis, or pupillary membrane lysis. He was also a pioneer in dacryocystectomy for chronic dacryocystitis and in congenital cataract surgery. His writings from 1716 onward repeatedly (and correctly) stressed that most of the patients with visual disorders required depression of the crystalline lens (for what he called glaucoma), as opposed to removal of an anterior membrane (which he called cataract).

**Conclusions:**

Woolhouse was a bold ophthalmic innovator and teacher who made major contributions which have lasted to this day. Although he did not admit it, he ultimately adopted much of the evolving understanding of the nature of lens opacities. However, his stubborn refusal to adopt the newer semantics has detracted from a full appreciation of his contributions.

## Introduction

Ophthalmology in Northern Europe progressed substantially from the Elizabethan era through the mid-1700s. Couching (depression) for cataract became common in England during this period. Surgery for congenital cataracts was reported in Britain in the decades after adult cases were performed.^1^ Surgeons learned that a cataract is an opacity of the crystalline lens and not an opacity anterior to the lens, as had been believed since antiquity.^2^ Angle-closure glaucoma was described in detail.^3,4^ Other procedures performed included posterior synechiolysis and dacryocystectomy.

**Figure 1.**
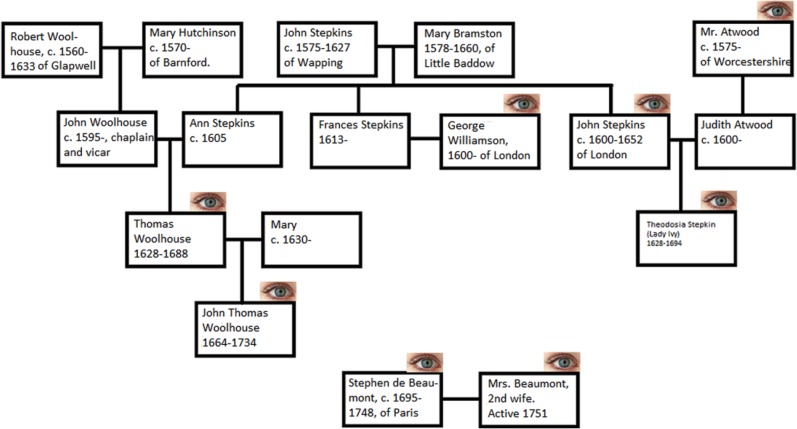
Family tree of the Stepkins and Woolhouse oculists.^5,6^ The eye icon represents oculists. Stephen de Beaumont was the nephew of John Thomas Woolhouse.

Ophthalmic healing during this period was a craft handed down from generation to generation within families. One such family was that of John Thomas Woolhouse (1664-1733/1734) of England. He wrote that he was 1 of 4 generations of fathers and sons who practiced eye surgery. Historians have known a little about his oculist father, Thomas Woolhouse (1628-1688). With today’s digital resources and databases, it is possible to tell the stories of at least 8 oculists in his family over 5 generations, spanning from 1600 to 1751 (Figure 1).^5,6^ We can also reassess the life and contributions of the most prominent oculist in the family, John Thomas Woolhouse.

## Mr Atwood of Worcestershire

The oculist patriarch of the family was one Mr Atwood of Worcestershire.^1^ Little is known about him, except that his daughter Judith married John Stepkins in Wolverley parish in 1625.^7^ Later Atwood oculists can be identified, although their relationship is unclear. In 1655, Mr Atwood, “an oculist of good fame” treated one Mr Alsop of Derbyshire.^8^ Author Samuel Johnson remembered that in 1711, “. . . my mother carried me to Trysul, to consult Dr. [Thomas] Atwood, an oculist of Worcester.”^9^ Trysull is about 12 miles from Wolverley.

## John Stepkins (circa 1600-1652)

Atwood’s son-in-law John Stepkins was consistently described as an eminent oculist. Stepkins had several medical influences. In 1606, his maternal uncle John Bramston (1577-1654) married Bridget, the daughter of Richard Moundeford (1550-1630), a prominent London physician.^10^ This connection sounds distant, but the families must have been close. When Stepkins’ wife died, he needed a guardian for his daughter Theodosia (known later as Lady Ivy), and he placed her with Mrs Moundeford.^11^ Mrs Moundeford herself sometimes treated the sick. In 1615, when an oculist was unsuccessful in treating a girl with a disorder of the eyes, Mrs Moundeford was called on to nurse her back to health.^12^

But it was Stepkins’ father-in-law who taught him the most: Stepkins “maried to Mrs. [Judith] Atwood, in Worcestershire, daughter of a very famous oculist, [of] whome this John learned much of his skill, tho’ he improved by practice extreamely.”^13^

Stepkins was known for a variety of eye waters and for performing congenital cataract surgery.^1^ Congenital cataract surgery had been performed by Ammār ibn ‘Alī Mawṣilī of Cairo in about ad 1000. Ammar couched a 30-year-old man from Kurdistan with a congenital cataract.^14^ (For this case, Ammar did not use the hollow needle he later devised to extract soft cataracts by suction.) Still, Stepkins performed the earliest congenital cataract surgery of which we are aware in England, as described by Robert Boyle:

The bare prospect of this magnificent Fabric of the Universe, furnished and adorned with such strange variety of curious and useful Creatures, would, suffice to transport us both with Wonder and Joy, if their Commonness did not hinder their Operations. Of which Truth Mr Stepkins, the famous Oculist, did not long since supply us with a memorable Instance: For (as both himself and an Illustrious Person that was present at the Cure informed me) a Maid of about Eighteen years of Age, having by a couple of Cataracts, that she brought with her into the World, lived absolutely blind from the moment of her Birth; being brought to the free Use of her Eyes, was so ravished at the surprising spectacle of so many and various Objects, as presented themselves to her unacquainted Sight, that almost everything she saw transported her with such admiration and delight, that she was in danger to lose the eyes of her Mind by those of her Body, and expound that Mystical Arabian Proverb, which advises, To shut the Windows, that the House may be Light.^15^

According to Boyle, the treatment was “a manual operation” performed “by my Ingenious Acquaintance, Mr. Stepkins.”^15^ Stepkins died and was buried on May 19, 1652 at St Mary’s Whitechapel church in London.^16–18^

## George Williamson (1600-After 1663)

George Williamson married John Stepkins’ sister Frances in 1633.^19^ Stepkins lodged with Williamson at the time of his death in 1652.^16^ A physician recorded that his 87-year-old father-in-law, David Tryme, from Wookey, near Bath, was unsuccessfully operated on by Williamson in 1663:

For, contrary to Dr. [Dawbeney] Turbervile’s Advice, (who counselled him to stay till he had been quite blind, when the Cataracts would have been ripe, and then he would not have questioned but to have helped him . . . by Couching of them). He hearing of one in London, in whose House Stephkin, the famous Occulist, formerly lodged (Father to my Lady Ivy, who also professed Eye-mending). This Fellow having seen Mr. Stephkin often perform that Operation, thought himself very able to do it, and set up for himself, (when his Lodger was dead,) and had a considerable Reputation for this Operation. This old Gentleman [Tryme] made a London-Journey at 87 Years of Age, or more; submits to this Fellow’s Cure; who without any kind of Preparation, of bleeding, or purging . . . performed the Operation, without any Regard, whether the Cataracts were ripe or no: This brought such a Flux of Humours first to that Eye, (for he had Couched but one) then to the other, afterwards to the whole Head . . . but caused him to lead a miserable Life the remainder of his Days . . . about a Year and some Months.^20^

## Theodosia Stepkins (Lady Ivy, 1628-1695)

Stepkins had a daughter named Theodosia, who through her marriage later became known as Lady Ivy (1628-1695).^1^ When she was a child, her mother died^11^ some time after the last sibling was born in 1631. Therefore, her father placed her in the London home of Mrs Moundeford, as noted above.^11^

Theodosia was notorious in her day and has been remembered for centuries, for several unfortunate episodes. In the early 1650s, she demanded alimony from her husband, Thomas Ivy.^11,17,21^ Theodosia made allegations of assault, infidelity, and infection with venereal disease, and Thomas Ivy made counter-claims of deceit, profligacy, murder of a chambermaid by poisoning, and conspiracy to murder him. Nonetheless, they reconciled in 1660, and Theodosia became “Lady Ivy” when her husband was knighted in 1661.^11^ When she sustained property losses in the Wapping (London) fire of 1682,^22,23^ she was hailed “. . . for her great pitty and Charity.”^22^

In 1684, leases Lady Ivy claimed established her ownership of property near Wapping were determined to be forgeries in a civil case.^11,24^ However, in the 1686 criminal trial for forgery, which was a capital offense, Lady Ivy was acquitted.^11,21^

In her day, Lady Ivy was also well known as an oculist.^1^ Unlike her father, her reputation as a healer appears to have been mixed. Lady Ivy, like her father, was known for applying eye waters.^1^ We have no evidence that she performed cataract couching.

## Thomas Woolhouse (1628-1688)

A chaplain named John Woolhouse joined this family of oculists by marrying John Stepkins’ sister Ann in London in 1627.^25^ John Woolhouse was a chaplain to the East India Company from 1619 until the year of his marriage. He was “ejected” as the Vicar of West Mersea, Essex in 1642, during the English Civil War.^26^ It seems that the minister never pursued a medical career. His oculist son Thomas Woolhouse wrote in 1675, “My father, being the first minister in Essex . . . that was plundered of any person whatsoever in Essex for his loyalty, was made incapable of giving me any other learning than reading the Bible . . .”^27^ Thomas Woolhouse entered the Colchester School in Essex in 1641 at age 13.^28^

Thomas Woolhouse served as the Page of the Presence (personal attendant) to King Charles II from March 19, 1673/1674 until the king’s death in 1685.^29^ On May 11, 1686, the appointment was renewed by the king’s brother and successor James II until Woolhouse’s death.^29,30^ According to his son, this royal service was in the capacity of an oculist.^31^ Thomas Woolhouse traveled to France to successfully treat Henry Howard (1655-1701), the seventh Duke of Norfolk, who had a “violent Ophthalmy and Defluxion on his Eyes.”^31^ The Duke was in France from March 24 to July 30, 1688.^32^ Woolhouse died and was buried on May 30, 1688.^33^

## John Thomas Woolhouse (1664-1734)

John Thomas Woolhouse was born in Halstead, Essex, and was baptized at St. Andrew’s church on December 23, 1664.^26,34^ In 1675, when he was 11 years old, his father wrote to the royal secretary,

As it is your favour to receive my son into your service we are ready to receive your commands for his coming to give himself wholly to your pleasure. Though he has not that complaisant humour the City brings forth, being always bred near Colchester till these two years, I hope you will find more genius in him to receive your commands than it is expedient to express, he being my son. He has had the experience of the want of learning . . . I have endeavoured to make him sensible thereby to quicken up his genius not to lose any opportunity.^27^

John Thomas Woolhouse claimed that he couched cataracts at age 13,^35,36^ presumably as an apprentice to his father. On April 26, 1681, a warrant was issued for him to have the first vacancy as Page of the Presence to Charles II,^37^ a position occupied by his father. Woolhouse matriculated at Trinity College in Cambridge in 1684 and graduated in 1686-1687.^26^ He studied eye surgery at Oxford.^35,38^ He converted to Catholicism and was therefore “disinherited by his father.”^39^ On his father’s death, it seems that he replaced his father as oculist to James II (who was exiled in December 1688 with the Glorious Revolution).

John Thomas Woolhouse left England after the revolution. In 1724, he wrote that he left due to the unhealthful London air: “the sealcoal air of London gave me a consumption in my youth which has been the chief cause of my fixing in this city [Paris].”^34^ But in 1698, he had written that he left to follow James II into exile:

Ever since the King my Master left England I have constantly attended him . . . as well in Ireland as in France, excepting about three years’ time . . . to study at Paris, Avignon, Montpellier, Pisa and Rome, and the late eight months I spent at Mons, Brussels, Maastricht, Aix la Chapelle, Liége, Cologne, etc., in the exercise of my profession of oculist . . .^39^

In Paris, Woolhouse became the surgeon at the Hôpital des Quinze-Vingts. He wrote in 1696 that he was the oculist to the exiled James II.^40^ He wrote that he could “give sight to those that are blind of cataracts, gutta serenas, pearls . . .”^39^

In 1698, Woolhouse requested to return to “my native country” given “my desire of marrying in England.” He promised not to make trouble with the state and “to treat all blind and sore-eyed curable poor people gratis.”^39^ He was told that his Catholicism and service to James II would make his return highly unlikely.^39^ We could not find any record of a wife or children for Woolhouse. Perhaps, he ultimately married in France, given that he referred to Stephen de Beaumont of France as his nephew.

The ophthalmic historian Julius Hirschberg reported that Woolhouse served as the oculist to William III,^35^ who died in 1702. Such service would be highly unlikely, as Woolhouse’s Catholicism and service to the exiled James II prevented him from even returning to England. In fact, Woolhouse was appointed oculist to James III by royal warrant on September 20, 1707.^41^ At this time, James III lived in exile in France but claimed the Throne of England as the son of James II. Known in England as “the Pretender,” James III was evicted from France in 1713.

Woolhouse knew about his oculist heritage. He wrote that he was 1 of 4 generations of fathers and sons who were oculists,^34^ that his family had many women oculists, and specifically mentioned Stepkins,^38^ Lady Ivy,^31,38,42^ and his father.

Woolhouse’s reputation extended to Moscow.^35^ In fact, Woolhouse demonstrated the cataract operation for Peter the Great in Paris in 1717, and the Tsar requested that Woolhouse teach a Russian pupil.^43^

### The private ophthalmology course

Woolhouse was teaching ophthalmology by about 1693. At times, his classes held 12 students from throughout Europe.^34^ He was still teaching as of 1725. His lectures were at least partly in French.^44^

The duration of training may have varied. One student, Christophe LeCerf, wrote, “Woolhouse is the only one in Europe who can give within one month a complete course on two hundred eye diseases with patient demonstrations and lectures. He lets his pupils operate as much as they wish to.”^35^ The lecture series of 1721 lasted approximately 3 months.^36^ Johannes Zacharias Platner, MD (1694-1747), and another prominent German student, Burkard David Mauchart (1696-1751), studied for 9 months in 1720.^35^

Proponents and detractors agreed that the training provided extensive hands-on experience. The English student, Benedict Duddell (circa 1695-circa 1765), stated, “. . . I have examin’d above a hundred blind in a Day at Paris, under Mr. Woolhouse in the Hospital of the Blind.”^45^ However, the French oculist Charles de Saint-Yves (1667-1736) wrote, “in Paris the eyes of poor people are sacrificed with impunity and without caution in order to have some material for apprentices who practice this operation for the first few months.”^35^

### The students of Woolhouse

Woolhouse’s best-known students were Platner, Mauchart, and Duddell.^46^ Duddell studied with Woolhouse in 1718^45^ and then settled in Hammersmith, near London. In 1736, Duddell offered one of the earliest descriptions of keratoconus.^47^

Woolhouse had kept his method of conjunctival scarification with beards of barley a secret, but one time when he was drunk, Mauchart wheedled the secret out of him. Mauchart “wrote an extensive manuscript about this course which he took with him and used occasionally in his later publications.”^48^ He hoped after his stay in Paris to study in Leiden, but the border was closed due to the bubonic plague.^48^ Mauchart eventually became a professor in Tübingen and died of an asthma attack in 1751, at the age of 55.^48^ Platner became a professor at Leipzig and rose to be the dean, before dying of an asthma attack at age 53.

We know of Woolhouse’s teachings from his journal articles and also from the books published by his students. LeCerf assembled letters and manuscripts of Woolhouse written between 1707 and 1716 and published them in French in 1717.^49^

Highlights of a manuscript containing notes from Woolhouse’s 1721 lecture series recorded by a student were published in 1934.^34,36^ The manuscript from the Royal Society of Medicine is entitled, “A Treatise of ye Cataract & Glaucoma. Dictated by Mons. Woolhouse, Occulist to ye French King, begun April 29, 1721.”^34,36^ We obtained this manuscript from the Royal Society of Medicine. Quotations from the 1721 lecture notes in our article use modern spellings.

Woolhouse’s teachings on cataract and glaucoma were published in 1745 by an anonymous student who made a written record of the lectures given in Paris “above twenty years ago.”^44^ The 1745 text includes at least some of the same anecdotes and teachings from the 1721 lecture notes.

The most extensive reviews of Woolhouse’s life and contributions were prepared by the great ophthalmic historian Julius Hirschberg and by RR James in 1934.^34,35^

Woolhouse’s career, with respect to glaucoma, can be divided into 3 periods. In the early period (before 1705), he emphasized hydrophthalmia, a condition of excess ocular tension treated with paracentesis. After 1705, he emphasized glaucoma in response to the new theory of the nature of cataract. He stated that glaucoma involved a palpably hard eye. Finally, by 1716, he acknowledged that couching was typically displacing the crystalline lens (however, he still refused to call this a cataract).

## A Brief History of Hydrophthalmia and Paracentesis

Before 1705, Woolhouse advocated paracentesis of the anterior chamber to relieve the excess ocular tension associated with a condition called hydrophthalmia (or hydrophthalmie). The diagnosis of hydrophthalmia began appearing in the literature by the end of the 1600s. The inspiration for this new thinking within European ophthalmic circles has never been properly explained. The name of the condition (hydrophthalmia) was new. Removal of aqueous had never been a therapeutic aim in Western medicine. The ancients recognized that cataract couching might result in loss of aqueous, but they looked at this as something to avoid, rather than a therapeutic end.^35^ Finally, the therapy involved a novel location of ocular entry. Some drained hydrophthalmia with needle puncture in the center of the cornea. This location had never been selected for instrument entry with couching, hypopyon drainage, etc. What could account for the sudden appearance of a novel disease and therapeutic strategy?

We believe the European surgeons were inspired by medical treatments in China and Japan, such as acupuncture. Direct sea routes between Europe and Asia had permitted increased opportunities for trade and interaction.

A Chinese text of ad 610, General Treatise on Causes and Manifestations of all Diseases (*Zhu Bing Yuan Hou Lun*), by Chao Yuan-fang, explained why “the eyeball protrudes”:

The eye is the [manifestation of the] essence-splendor of the yin and yang [qi] of the viscera and the bowels . . . [and the] indicator of the liver. Whenever . . . wind-heat or phlegm-liquid collect in the viscera and bowels, yin and yang [qi] are not in harmony, the liver qi accumulates and generates heat. The heat rushes to the eyes and causes the pupil to have pain . . . and hence causes it to protrude.^50^

According to *Essential Subtleties on the Silver Sea* (*Yin-hai jing-wei*), compiled in the 14th or 15th centuries,^50^ eye protrusion was subdivided into 4 subtypes and was still believed to be due to heat in the eyes. In this era, the treatment was to puncture the eye to release water. For instance, “pearl-like black shade” involves tearing and eye pain until “. . . the water sphere protrudes and forms a black shade resembling a pea or a pearl . . .”^50^ The upper lid bulges and it is difficult to move the eye. The contralateral eye can eventually become involved.^50^ The treatment is to “Pierce the black shades one by one from the side with a fine-tipped needle, and tear them open. After the bad water that each contains has flowed out, [the protrusion] will be leveled.”^50^

“Painful crab eye” was a condition which “arises from the pupil” and “resembles a pea or a pearl.” The text notes “The root of the shade is small, while its sprout is big.”^50^ The translator suggests that this image suggests an incarcerated iris prolapse.^50^ The eye is red, painful, and hard to open. ^50^ Again, the doctor will “Pierce with a fine needle tip to let the bad water flow out.”^50^ Then “the prominence will be leveled.”^50^

In “helical protrusion,” there is eye pain, and “the pupil in the center [of the eye] gradually changes to a bluish white. Suddenly it bulges out.” ^50^ Once again, “one must puncture with a fine-tipped needle in the direction of the center of the pupil, and insert the needle one-half fen [about 1.5 mm], and let the bad water flow out. This way [the protrusion] will become level.”^50^

In “protrusion of the eye,” “the qi-poison from the five viscera attacks the pupil.”^50^ Again, there is tearing and possibly pain. If the lesion is

purulent and protrudes one cun [about one inch], then one must puncture with a fine needle tip to let the bad water come out. Only then will the pain stop, and the protrusion of the eye will be reduced to its [normal] level.^50^

Thus, in Chinese medicine, any sort of painful, inflamed corneal protrusion (anterior staphyloma or Descemetocele), or perhaps even proptosis, whether or not accompanied by hypopyon or a change in pupillary color, was treated with needle puncture to drain water (aqueous).

When Kovacs and Unschuld write that the *Yin-hai jing-wei* does not mention acupuncture,^50^ they imply that the Chinese considered neither couching nor paracentesis a form of acupuncture. It is accepted that acupuncture was developed in China, and then couching for cataract was subsequently imported to China from India.^51^ However, Fan writes that “After the Tang dynasty [ending 907 AD], Chinese physicians regarded couching for cataracts as an element of acupuncture.”^51^ In other words, the Chinese appropriated a foreign technique within a traditional Chinese medical framework. In any event, European physicians who imported paracentesis from China may have viewed it as a form of acupuncture.

There is circumstantial evidence that the first European author to describe anterior chamber paracentesis for “hydrophthalmia,” Michael Bernhard Valentini (1657-1729) of Germany, was inspired by Asian medical techniques. Valentini corresponded with Andreas Cleyer, who edited *Specimen Medicinae Sinicae*, which introduced the idea of acupuncture to many Europeans.^52^ Published in Frankfurt in 1682, the book was based on the work of Michael Boym, a Polish missionary who had spent time in China at various times between 1643 and 1659.^53^ While in Batavia, Cleyer obtained Boym’s manuscripts. The work illustrated acupuncture points and pathways and translated the Chinese concept of *qi* as *spiritus*.^53^ The work also implicated “humidum radicale” (radical moisture) in disease.^52^

The term “acupunctura” first appeared in a 1683 treatise on Japanese medicine by Dutch physician Willem ten Rhijne (1649-1700).^54^ ten Rhijne had studied medicine at Leiden and Angers (France), and beginning in the 1670s, practiced in Deshima (Japan), and traveled to Edo (Tokyo).^53^ ten Rhijne translated the Chinese concept of yin as *humidum radicale* and yang as *calidum innatum* (“innate heat”).^53^ He mentioned that acupunctura could be used for “ophthalmia” (eye inflammation) and for “*oculorum lippitudini ac suffusion* [ocular inflammation and suffusion].”^54^

In 1686, a major excerpt from ten Rhijne’s work, the portion on gout, was translated into English.^55^ Acupunctura was rendered as “acupuncture,” thus coining the English term: “They cure the Gout speedily and easily . . . Among themselves they have, by the guidance of China, adapted a two-fold method of Cure to the foresaid Diseases, namely, Acupuncture, and burning with their Moxa.”^55^ Moxibustion was a related Chinese therapy of burning dried vegetable matter (called moxa) placed on specific points of the body. *Humidum radicale* became “radical moisture,” although its imbalance was not central to the pathology of gout:

. . . the China Physicians say, Our Bodies are governed by 3 things, i.e. by the innate Heat, the radical Moisture and Spirits, which they hold to be the Vehicle of the Heat, and the Lungs . . .^55^

The editor appended the experiences of several notable persons who had moxibustion for gout at the Hague, and noted that ten Rhijne’s son was advocating moxibustion at Utrecht.^55^ The editor proposed that acupuncture removed water from the tissues. For instance, “Hydrops Anasarca, or, A Dropsie in the Flesh” was cured by

. . . getting out intercutal Water by Acupuncture . . . Take an ordinary Needle (such as Tailers use) and prick the Skin with it in the most oedematous place . . . the Water will burst out drop by drop out of every such little hole . . . till the Swelling round the prickt places do vanish.^55^

The editor noted that aqueous could escape when couching for cataracts: “. . . lest the Watry Humour should run out, after taking out of the Needle. M. Valentin, a great Oculist, observed this.”^55^

Valentini, born in Giessen, Germany, spent time in France, England, and the Dutch Republic.^53^ He was familiar with the work of both Cleyer and ten Rhijne. Back in Giessen in 1686, Valentini published a work recommending moxibustion for gout, which he dedicated to Cleyer and in which he cited Cleyer’s *Specimen Medicinae Sinicae*.^56^ Valentini corresponded directly with Cleyer.^53^ In 1704, Valentini published *Museum Museorum* in Frankfurt^57^ which contained reports of the East Indies, including letters on botany dating from 1683 to 1687 addressed to ten Rhijne, from Georg Eberhard Rumphius, who was working in Batavia.^53^

Valentini entitled his report “Hydrophthalmia puncturâ acus percurata [hydrophthalmia cured by acupuncture].”^58^ We provide complete translations for some of these descriptions because to the best of our knowledge they have not been published in English. Valentini reported,

. . . observations communicated by the learned gentleman Wessem, the physician of Frankfurt, who is most famous everywhere for curing various ailments of the eye . . . The first is the cure of hydrophthalmia [*hydrophthalmiae*], for curing which entirely he had recommended the eradication of the eye. Yet he tried at first to see whether to achieve something by perforating the eye, which proceeded so well that, once the humor was entirely evacuated, things had been applied internally, and a diet was properly prescribed, the illness was entirely overcome.^58^

Valentini’s report includes the first known use of the term hydrophthalmia and, in the West, of therapeutic paracentesis to remove aqueous. Of course, paracentesis had long been used to drain hypopyon, or inflammatory material, from the anterior chamber. For instance, French surgeon Ambroise Paré described the drainage of hypopyon in the 16th century.

Paracentesis for hydrophthalmia was next championed by the Dutch surgeon, Anton Nuck (1650-1692) in 1690.^59,60^ Nuck’s descriptions seemed reminiscent of the Chinese descriptions of protrusions from the eye. Immediately after reviewing Valentini’s case, Nuck related,

I was able to observe a no less notable case in a most respectable woman of the Hague, the bulb of whose eye I saw to be not so distended, after serious inflammation of the eye [*ophthalmia*] had developed, but to be filled with three excrescences like horns. Having tried various things in vain, it was decided in the end to pierce one of the swellings (which I suspected to be full of aqueous humor) with the help of a needle. This was done with such success that water flowed out in abundance, and each of the swellings was rendered smaller . . .^59^

Nuck described another patient with hydrophthalmia^59^:

The youth Bredan, being around 24 years in age, after rather stubborn ophthalmia, and most serious pains in his left eye, had gradually lost sight in it, and the pupil was rendered a more obscure colour, because of rather turbid aqueous humour, which was flowing in rather copiously, and the bulb of the eye had started to become so extended that, within a few months, it had entered into hydrophthalmia of prodigious magnitude; the eye, bursting forth beyond its orbit, and the eyelid being unable to shield it any more on account of the magnitude of the mass, rendered it more like that of a cow than a human . . . I knew from sure experimentation that to extract the aqueous humour, and to return it to its former and natural size, the eye would have to be fortified later with an artificial eye . . . thus, having brought together the eyelids, blocking out the light, in the middle of the pupils (where the vessels are minima) we perforated the transparent cornea, with the help of our needle; and the bulb of the eye, via aqueous humour flowing out in streams, was immediately rendered about a fifth smaller in its circumference . . . on the following and fourth day we decided that a third perforation should be deferred, at which time, as on the second day, we saw it distended from the internal water applying pressure: therefore for a third time the cornea was punctured and, being harmed by a larger wound, it poured out a more copious amount of water . . . on the tenth day, having punctured the cornea (by inserting a thinner tube), we drew out as much aqueous and vitreous humour by sucking it out, so that it (the eye) differed little from its natural size . . .

Nuck apparently performed paracentesis through the center of the cornea. He claims to have removed vitreous as well by suction on the 10th day (although it is difficult to see how vitrectomy could be accomplished with a point of entry anterior to the lens). Nuck’s report is the first of therapeutic vitrectomy of which we are aware.

Woolhouse and his students were explicit that paracentesis had been imported from Asia. Woolhouse taught that paracentesis was performed in the “Indies” and that he had seen it performed by the English oculist Dawbeney Turberville, MD (1612-1696):

. . . So if ye only certain remedy ye has ever yet been found, from Hippocrates time, to this day to hinder ye progress of cataracts is to let out ye gross watery humour by ye operation of ye paracentesis, now in use in ye Indies as I have been credibly informed and which I have seen practiced in England by Dr. Turbeville, . . .^36^

This information is also in the 1745 edition of his lectures.^44^ It is not clear from this passage whether by “ye Indies” Woolhouse meant the West Indies or Asia. In our research, we have found that paracentesis was performed in Asia, but not in the West Indies.^61^

Mauchart reported that Turberville learned of the procedure from a ship captain who had spent 15 years in Peking. According to Mauchart, it was Woolhouse’s father who observed Turberville performing the procedure, and the younger Woolhouse performed it twice in 11 years in France and Ireland.^62^

We are not aware of specific reports of Turberville reporting paracentesis for elevated ocular tension. The closest case is one he recorded in December 1684, which had occurred about 6 years previously (1678), involving medical treatment for a young man with “an eye as big as a hen’s egg . . . from thin humors fallen on the eye, and extending its coats”^63^ Thus, just before the term “acupuncture” was recorded, Turberville does not seem to have been using ocular paracentesis. In 1685, Turberville called this *Oculum Bovinum* or *Oculi Hydropem*.^63^ The journal index recorded this as a case of *Oculi Hydrops*.^63^

Subsequent authors confirmed the origin of paracentesis in Asia and its equivalence (in their minds) with acupuncture. A medical dictionary mentioned Valentini’s paracentesis in the article on acupuncture:

Acupuncture . . . a method of curing many diseases by pricking several parts of the body with a needle . . . This is practised every day by the Chinese and Japanese . . . We sometimes also find mention of an Acupuncture practiced in Europe; but this amounts to no more than the perforating or opening a part, e. gr. the cornea, with the point of a needle; which has been done with good success, for the cure of an hydrophthalmia and hypopyon. Valentin.^64^

Of course, Valentini’s language (“puncturâ acus”) evoked the Asian procedure.

Under the heading “Hydrophthalmia,” Velpeau noted that after failure of conservative measures

. . . I can see nothing more rational than paracentesis of the eye . . . Though used in Japan and China for some centuries, and practiced by Tuberville and Woolhouse, this remedy does not appear to have been formally proposed by any one for hydropthahlmia, before Valentini, Nuck, and Mauchart.^65^

Thus, anterior chamber paracentesis for ocular protrusion was performed in Asia initially, and early European advocates of the procedure knew of this history and were inspired by the Asian techniques.

The Europeans modified the Asian understanding of the conditions treated by paracentesis in several ways. First, the European authors did not think of the eye as a single compartment because they knew of the crystalline lens. Thus, hydrophthalmia came to be thought of as excess fluid in the anterior chamber, the posterior chamber, or the vitreous compartment. As the internal derangements of the separate compartments of the eye rose in importance, the protrusion of a part of the eye (staphyloma) or of the eye itself was not as uniformly emphasized (but did not disappear).

As we show in the descriptions below, elevated intraocular tension, if not universally mentioned, seems to be implicit in many cases of hydrophthalmia. Whether the ocular tension was gauged by palpating the eye or by the patient’s sensations and the appearance of the eye is not explicitly stated. Today, of course, we understand that elevated intraocular pressure does not appreciably increase the size of the eye, unless it occurs during childhood development (buphthalmos), or if the ocular coats have been damaged by infection or inflammation.^66^ Hydrophthalmia continued to be the standard term for congenital glaucoma until the 1950s.

We are left with the question of why hydrophthalmia descriptions continued to mention ocular prominence, at least to some degree. In part, there may have been inertia and a willingness to accept traditional definitions. The authors did not have any quantitative metric, such as Hertel exophthalmometry. Some authors did distinguish between an appearance of prominence and true prominence. For instance, Middlemore distinguished between “projection, or a sort of *appearance* of projection.”^67^ In the absence of effective treatments, high pressures or inflammation might have eventually led to scleral or corneal breakdown and true ocular protrusion (staphyloma or Descemetocele).

We can see some hint of the evolution of the term hydrophthalmia in Woolhouse’s rather incomplete descriptions. Woolhouse cited Guillemeau and Nuck and advertised early in his career that he, too, performed surgery for hydrophthalmia. The term *parakentesis* had been used in antiquity by Galen to describe puncture of the eye when depressing the cataract. Ocular paracentesis became promoted and identified with Woolhouse to such a degree that a contemporary called it “that bold operation of Woolhouse.”^68^ It must have been an important part of his practice because Woolhouse stated that he was preparing an ophthalmic treatise, which would cover treatment of (in order): hydrophthalmia, cataract, and gutta serena. The language suggested that his readers would be unfamiliar with the term: “a certain malady of the eye, which he calls hydrophthalmia, dropsy, in the body of this organ of vision.”^42^

Later in 1696, Woolhouse noted that he had treatments for 22 diseases, the first of which was cataract, and the second of which was hydrophthalmia: “The puncture of the eye by ocular paracentesis, an instrument invented by Mr. Woolhouse, a new operation done in hydrophthalmia, or dropsy of the eye.”^69^

In 1703, Woolhouse provided more detail:

The ophthalmic paracentesis, the puncture, pertusion or perforation & piercing of the eye, not only in amaurosis and disturbance of the ocular humors; but in the swelling of the globe of the eye, and distention of its membranes which often push the eye out of its orbit, burst it, and cause many deplorable accidents, in addition to loss of sight, which just happened, it is said, to the first President of Rouen, for lack of the above operation. Our ophthalmologist is the first who put into practice this operation in hydrophthalmia or dropsy of the eye, when the humor (principally aqueous) errs in quality, blurring vision, and exceeds in quantity the aqueducts of the eye, being dilated, etc.^70^

Note that Woolhouse contrasts swelling of the globe with “amaurosis” (which involved a normal-appearing eye), and with a mere “disturbance of the ocular humors” (presumably without globe enlargement), but recommends paracentesis for all of them.

In his discussions of cataract and glaucoma after 1705,^71–78^ Woolhouse typically failed to mention hydrophthalmia.^71–75,77–78^ Hydrophthalmia, when mentioned during this period, was still treated with paracentesis.^76^ In his treatise on glaucoma, Woolhouse briefly mentioned Galen’s use of the term “Parakentesis, a punction,” and noted that the term had been appropriated for the “operation for the dropsy, called in English, tapping.”^44^ Despite his de-emphasis of *hydrophthalmia* after 1705, Woolhouse still advocated paracentesis occasionally:

. . . there is frequently such an influx of humors of ye morbifick matter fills not only ye second compartment of ye eye but passes through ye apple, to ye first region and having at last no farther room, distends ye globe prodigiously, and all ye remedies in ye desperate case is to perform in ye eye so affected ye same operation as in ye hypopyon or in ye empyema [i.e. paracentesis].^36^

Woolhouse’s student Platner described hydrophthalmia, involving ocular swelling and pain due to poor outflow of aqueous in the veins, and its treatment with ocular paracentesis.^44^

Woolhouse’s student Mauchart provided more complete descriptions of the various types of hydrophthalmia, all treated with paracentesis, in 1744. When the expansion was anterior to the iris, the anterior chamber was described as deepened. These cases might correspond with pigmentary glaucoma, angle recession glaucoma, resorbed cataract, or Descemetocele. According to Mauchart,

Diagnosis of hydrophthalmia: the successive increase of the sphere of the eye, increasing its natural dimensions by a third, half or the same size again: swollen tension; the cornea raised and protruding more than is usual; yet the iris being deeper and more removed from the internal surface of the cornea; the pupil unmoving, sometimes larger, otherwise narrower and thinner; the sight is at first unimpaired but subsequently weaker and more obscure; especially when, as frequently happens, the dark clouding of the cornea and the murkiness of the aqueous humour occur together; for some there is a dull stretching pain around the base of the eye, which although almost continuous is nevertheless very mild; in others it is much more serious along with a headache on the same affected side, stupor in parts of their face, and sometimes chest pain of the whole side, toothache and insomnia. In addition, as a result of the additional increase in mass, exophthalmia, illacrimation and ectropion . . .^79^

A second type of posterior hydrophthalmia due to vitreous expansion was described by Mauchart and others. Many features resembled angle-closure glaucoma:

Swelling and preternatural increase of the vitreous humour likewise notably increases the dimension of the eye and gives it a firm tension [duramque infert ei tensionem]; yet if this happens without the accompanying increase of the aqueous humour, one can then easily see, as Woolhouse testifies, the rim of the vitreous humour elevated around the crystalline lens, covering it with a shadow, creating upwards strabismus, producing extraordinary firmness [duritiem] for the bulb, and introducing a dull pain along with a notable loss of vision. Then the iris without doubt nears the cornea and assumes such a convex shape but one that is wholly diseased.

But if the preternatural increase of the aqueous humour and of the vitreous humour occur together, as it is agreed can and tends to happen a priori and according to the observations of Nuck in his Sialographia Nova, p. 123. the diagnosis is more difficult unless, on account of the excessive mass growing too quickly, and the outstanding firmness [duritie] and strabismus, one may prophesy the concurrence of both defects.

Nor does this difference matter much in terms of the cure. For in the end, paracentesis duly employed in the sclerotica whitewashes two walls from the same pot.^79^

This Latin idiom is comparable to “kills two birds with one stone.” Thus, in this view, with hydrophthalmia due to vitreous expansion, prominence of the globe is not a prominent feature. Rather, the condition involves pain, vision loss, a palpably hard eye, and a convex iris which approaches the cornea—all findings consistent with angle-closure glaucoma.

François Boissier de la Croix de Sauvages of France noted several types of hydrophthalmia. The first type, also known as “vitreo-pupillary ophthalmy,”^78^ involved eye pain and “the pupil is much more dilated.” Prominence of the eye was less noticeable, and

“This [condition] in the beginning is with difficulty distinguished from an incipient common cataract, and also from the cataracta glaucoma; but seeing that no opacity of the crystalline lens comes on . . . thus it is known from other diseases.” The disease was due to excessive vitreous causing “a pressure of the retina.”^80^

Boissier proceeded to summarize the types of hydrophthalmia described by Mauchart due to either excessive aqueous or vitreous, or both. Excessive vitreous involved “a particular hardness and turgid tension . . . the iris convex, approaching nearer to the Cornea; the pupil more dilated than usual and altogether immoveable.”^80^

In 1833, English surgeon William Lawrence described a case of “hydrophthalmia” which was consistent with uveitis and a resorbed cataract. Lawrence wrote of “a case of inflammation of the eye, accompanied with enlargement of the anterior chamber.” The 25-year-old patient developed eye inflammation serving as naval officer in the Mediterranean. After 1 or 2 years, Lawrence noted,

At first view the globe appears enlarged, but I can discover no increase of size, except in the anterior chamber, which contains about three times the usual quantity of aqueous humor . . . The iris and pupil are nearly natural, and move well. The lens is opaque.^81^

Lawrence treated this case with anterior chamber paracentesis.^81^ It is interesting that Lawrence noted that the initial appearance of a prominent globe might be deceiving.

In 1835, Middlemore mentioned the term hydrophthalmia but preferred the phrase “dropsy of the eye,” which could be due to excessive aqueous, vitreous, or “subchoroid dropsy.”^66^ He recommended evacuation of the excessive fluid.^66^ The type with excessive aqueous might involve a deeper anterior chamber. But Middlemore also noted that the pupil could be small, with “the iris convex anteriorly.”^66^ These cases could have been pupillary block associated with posterior synechiae. Middlemore advised that after puncture of the cornea to release fluid, “The practice of maintaining the patency of the aperture which has been made, by the introduction of a tent, as advised by Mauchart, cannot be sufficiently condemned.”^66^ Dropsy of the vitreous involved globe prominence, “the iris is nearly in contact with the cornea; the pupil expanded and motionless . . . the sense of tension of the globe is much increased.”^66^

Some 18th and 19th century authors only used 1 term, either hydrophthalmia or glaucoma, at a given point in their careers. Authors who used both terms simultaneously might have reserved hydrophthalmia for cases with a dark pupil and glaucoma for cases with a green or gray pupil.

## The New Theory of Cataract

After 1705, Woolhouse de-emphasized the term hydrophthalmia, and for the most part, appeared to replace it with the term glaucoma. This change occurred as he attempted to rebut the new (and correct) theory of cataract, which held that the structure displaced by couching was the crystalline lens, rather than an opacity anterior to the lens. Woolhouse insisted on describing disorders of the crystalline lens as glaucoma because several ancient authors, including Galen, had used this term to describe a disorder of the lens.^3,4,82^

Prior to the 18th century, the cataract which was displaced by couching was believed to be a membrane anterior to the lens.^2^ The lens itself was considered the seat of vision, as we might view the retina today.

In contrast, some French observers had proposed that a cataract was in fact an opacification of the lens in the 1600s. For instance, the idea was articulated by Antoine Le Grand (1629-1699) and translated into English in 1694:

Those that have a Cataract Couch’d, discern but obscurely all visible Objects; whereupon that they may the more clearly and distinctly see them, they make use of Convex Glasses . . . a Cataract is not any Skin (as hath been long believed) growing between the Chrystallin Humour and the Uveous Tunicle, which may be taken off by a Needle, and drawn down to the inferiour part of the Eye, but that it is the Chrystallin Humour it self, which in tract of time grows flaccid and weak, and is separated from the Ciliary processes, as an Acorn when ripe, is easily separated from its Cup, forasmuch as it is removed with little or no trouble, and deprest to the very bottom of the Vitreous or Glassy Humour, a small part, in the mean time, of the said Vitreous Humour succeeding in its place. The Cataract therefore being thus taken away, the Chrystallin Humour also must of necessity be taken away, or at least be rendred more plain, or less convex, whereby it comes to pass that the Rays proceeding from all points of the Object, are not sufficiently broken or made bending, so as to be united in the Retin, when they arrive there: Whence the Vision or act of Sight must needs be confused. To which infirmity the Chrystallin Convexity only gives relief, as causing the Rays which before were divergent to become convergent, and to enter the Eye with such a disposition.^83^

However, this idea did not initially achieve widespread circulation among surgeons.^34^ In the early 1700s, the idea reemerged in Paris, and this time, the correct understanding of the anatomic structure displaced by couching became generally accepted. On April 7, 1705, a young French physician, Michel Brisseau (d. 1743), was skeptical of the ancient (but prevalent) teaching and therefore couched the cataract of a soldier who had died the previous day. Brisseau then determined by dissection that the crystalline lens had been displaced into the vitreous. Brisseau’s observation was read in the French Royal Academy of Science on November 18, 1705.^34^

The French oculist Antoine Mâitre-Jan (1650–1730) published several relevant observations in 1707.^34^ When the cataract was subluxated into the anterior chamber during couching, he noticed that it was thick and not a thin membrane. Mâitre-Jan noted the cataract to be an opaque crystalline lens at autopsy in patients who had been couched and in others who had not.

On February 20, 1707, Saint-Yves extracted through a corneal incision fragments of a crystalline lens which had spontaneously dislocated forward, producing inflammation.^34,84,85^ This cataract extraction was performed in the presence of the French surgeon Jean Méry^86^ and succeeded in relieving the patient’s pain.^85^

A second extraction of a lens which had subluxated into the anterior chamber was performed by the French surgeon Jean Louis Petit (1674-1750) on April 17, 1708 in the presence of Saint-Yves and Mery.^34,86^ A “skillful English oculist,”^86^ believed to be Woolhouse,^87^ was consulted. He proposed to meet the surgeons at the Academy of Sciences, to demonstrate that a membranous cataract had been extracted, but he did not come. However, on the appointed day, the patient and the extracted structure were examined, and the observers agreed that the patient could see with convex spectacles, and that the crystalline lens had been extracted.^86^ Woolhouse, in writing about the event, claimed that the patient was the one who failed to appear on the appointed day.^85^ This case probably did the most to convince skeptics that the lens was not the seat of vision (as had long been proposed) because the patient was able to see afterward.

Saint-Yves described 3 cases of cataract extraction, including the 2 above and a third in 1716. Saint-Yves wrote that he performed this procedure whenever the lens became subluxated into the anterior chamber, writing, “I have formed many of these Operations.”^85^

Woolhouse also accepted the extraction of intraocular opacities, although he used the term *cataract* for a membranous opacity, and the term *glaucoma* for an opaque crystalline lens. His precise description suggests that he may have performed this procedure routinely:

The tenth operation is when the cataract or glaucoma has passed into the pupil, between the cornea and the iris. It is called extraction of the cataract or glaucoma, and consists in a longitudinal section of the cornea, a little below the opening of the iris. The reason of making it here is, that as there will remain a dark cicatrix after the cure, the sight would be obstructed by it, more or less, if it traversed the front of the pupil.

To perform this operation, the patient must be placed in the shade, where the pupil may be as much as possible distended: then planting the glaucomatic needle in the cornea, a line’s distance from its outward circle on the temple side, and making it come out on the nasal side a line’s breadth also from the circle; with a lancet made for the purpose, that must be no broader than a cataract needle, and cuts only on one side, make an incision according to the direction of the needle, the whole length of its entrance. The patient must be turned up on his back in the instant, without pillow or bolster, and the cataract or glaucoma drawn out of the first chamber of the eye, with an instrument made also for the purpose.^43^

## A Brief History of the Term ***Glaucoma***

Woolhouse’s principal objection to the new theory of cataract was that some ancient authors had used the term glaucoma for disorders of the crystalline lens. Woolhouse was being selective in his reading of the ancients. The term glaucoma was used more broadly during antiquity to refer to the light-colored (green or gray) pupil, and it was just a fraction of the authors who ascribed this color to a disorder of the lens.^3,4,82^ In Woolhouse’s defense, this understanding was found in the writings of prominent authors, such as Rufus of Ephesus and Galen.^3,4,82^

Just before Woolhouse, glaucoma was not commonly discussed. Glaucoma was just 1 of 113 eye diseases in Guillemeau’s treatise and was not even mentioned in less comprehensive texts.^88^ Guillemeau’s description of glaucoma was typical of the period: “. . . glaucoma is properly used when the crystalline humor is dry and thick, and the color of it is green . . .”^88^

Further evidence that “glaucoma” was not commonly written about comes from the Early English Books Online database, which contains 44 000 books published before 1700. In this database, just 39 books contained the text “glaucoma,” and only 2 of these described the crystalline lens as hard.^89^ Jean Riolan, the elder, wrote of glaucoma^90^:

. . . or if the crystalline humour is changed into a grey colour (albeit with the admixture of white and green), which blight is called *glaucosis* or *glaucoma*, the surface of the crystalline humour is hardened and overcome by dryness, and that which should be bright, clear and even becomes uneven. Under glaucoma everything is seen by us obscurely, and as if through shade: light is not seen, which occurrence distinguishes it from a cataract (*suffusio*). Why does *glaucosis* come from old age? Because it is wrinkled by dryness, a condition that is incurable, just like other diseases contracted from excessive dryness.

Similarly, Jean Riolan, the younger (1580-1657), wrote,^91^

The thickness and hardness of the Chrystallin Humor is properly termed Glaucosis or Glaucoma, because the color thereof resembles that of an Owles Eyes: it proceeds from a cold and dry distemper, and is therefore familiar to aged Persons.

It seems most likely that the characterization of the crystalline as hard was offered as a theoretical property, no more amenable to clinical assessment than whether the humor was dried. Nothing in either statement suggests palpation of the eye.

Woolhouse initiated a bitter debate with advocates of the new theory. In a letter read at the French Academy of Sciences in April 1707, he protested that the ancients had always used the term *glaucoma* for disorders of the crystalline lens. It appears that Woolhouse plucked an esoteric condition from obscurity and thrust it squarely into the debate over the nature of cataract. When his 1707 letter and his other objections were collected, the term *glaucoma* was in the title of the resulting 379-page book.^48^ The 12-page review of Brisseau’s 1706 work, *Nouvelles Observations sur la Cataracte*, did not even mention glaucoma.^92^ But after Woolhouse’s objections were raised, Brisseau’s 1709 response had the word glaucoma in the title.^93^ Glaucoma also was in the title of the responses of French surgeon Jean Mery in August 1707 and 1708^86^ and subsequent texts.^94–96^ Glaucoma did not become a ubiquitous diagnosis until after 1850 when the ophthalmoscope permitted visualization of the characteristic optic neuropathy,^97^ but Woolhouse had reinforced its importance in the minds of scholarly oculists.

## A Brief History of Diagnostic Palpation of the Eye

In addition to writing that his opponents were simply couching “glaucomas,” Woolhouse did something else quite curious. He taught that glaucoma involved a palpably hard eye. Was he the first to do so? What is the history of palpation of the eye?

Diagnostic palpation of the eye was potentially performed in antiquity to assess the maturity of cataracts. The idea was that a cataract started as a liquid and when mature would solidify. This idea was first expressed by Celsus:

And in the cataract itself, there is a certain development. Therefore we must wait until it is no longer fluid, but appears to have coalesced to some sort of hardness.^82^

Hirschberg interprets this passage to imply actual palpation of the eye.^98^ Pressing on the eye and looking at the movement of the cataract within the eye permitted assessment of its suitability for couching. Writings of the 17th century show that this teaching survived and was indeed interpreted as requiring actual palpation of the eye.

Lazare Rivière (1589-1655) noted,

If this Operation be, when some part of the Suffusion floweth down (if the eye be compressed) and appeareth more large, and after returneth to its former station and figure, it is not successful; because the Cataract is not yet ripe, but thin and crude: But if by a compressing with the finger there is no change of the shape and figure of it; it is then ripe, and may be couched with a Needle.^99^

English ophthalmologist Richard Banister was the first European author to suggest that firmness of the eye to palpation indicated that visual loss could not be restored with couching. Banister’s *Breviary* of 1622 stated that a *Gutta Serena* was unlikely to be cured:

if one feel the eye by rubbing upon the eyelids, that the eye be grown more solid and hard, then naturally it should be . . .^88^

It is interesting that Banister’s Breviary was republished in 1706, the year before Woolhouse’s April 1707 letter. Woolhouse did cite Banister.^88^

Woolhouse combined the concept of the hard eye, described by Banister and implicit in the hydrophthalmia concept, with the disorder of the crystalline lens, termed *glaucoma*, of the ancients. In doing so, he forever changed ophthalmologic language. As early as April 1707, Woolhouse wrote that the finger could determine whether the crystalline lens was hard^48^:

But I have found an infinity of glaucomas of the crystalline humor, where the vitreous and aqueous humor were healthy. In these one feels a hard crystalline, resisting the finger, which distinguishes them from true cataracts, and no author, that I know, has remarked on the following symptoms and diagnostics that my late father, celebrated English oculist, taught me, and which I never fail to see: a true glaucoma comes ordinarily little by little to the two eyes over time, after severe headaches, after blows to the eyes, after long illnesses, or with advanced age.

The mention of the finger demonstrates that this is not a theoretical concept but a physical property which could be clinically assessed through palpation of the eye. Any doubt is resolved by Woolhouse’s lectures. The following description suggests what today would be termed *phacomorphic glaucoma*:

But cataracts are in this different from glaucomas. Ye cataracts adhere to ye inside of ye fringe of ye iris and are as it were glued to it. And looking on one side, one may see its threads above or below or only right or left side.

But ye glaucoma adheres not to ye Iris unless it be quite unsheathed and fallen out of its calix of ye glassy humor, which all very ripe and hard glaucomas will do in process of time and thereby imitate so perfectly a true cataract if there will be no distinguishing ye one from ye other by a sudden inspection. And then ye feeling is ye only way to have a true knowledge thereof, for such a hard and dry glaucoma reclining upon ye inside of ye iris dilates ye apple of ye eye and makes it immoveable, and without spring if it chance to be pushed upon ye hole in ye iris as a stone in a sling. But if it happens to fall upon ye iris with ye pupil is well nigh shut then it hinders ye pupil from opening and dilating itself, and ye forepart of ye eye will feel harder to ye finger and reclining ye head backwards, and rubbing of said eye you’ll perceive ye crystalline humor return with a perceptible noise.^35^

He noted that a glaucoma grown “older and harder” can press against and hinder the motion of the iris.^43^

These passages from Woolhouse were not needed to rebut the new theory of cataract. They explain how “glaucoma,” a term for disorders of the lens, came to be attached in the modern era to a chronic optic neuropathy associated with ocular hypertension. Woolhouse observed, correctly, that a swollen lens can impair motion of the iris and can lead to a palpably hard eye.

One might argue that it was inevitable that this linkage would occur. After all, because the crystalline lens was considered the seat of vision, its disorders were considered incurable. Moreover, the optic neuropathy resulting from ocular hypertension is incurable. But this presumed inevitability suggests a paucity of imagination influenced by familiarity. After all, there could be new coinages, such as hydrophthalmia. Moreover, disorders of the optic nerve were also considered incurable in antiquity. Hunton’s translation of Guillemeau lists many terms for disorders of the “nervus opticus” or “sinew of sight”: *amaurosis, obfuscatio, gutta soerena, parorasis, hallucinatio, calligatio, symptosis, aporrexis, abruptio, paremptosis*, and *coincidentia*.^100^ As we have seen, *gutta serena* was in fact used by Banister in his Breviary to describe the palpably hard eye with incurable blindness.

The precise influences on Woolhouse cannot be known. Woolhouse is known to have read Banister’s Breviary and also cited the author who republished the Breviary in 1706 and 1710.^88^ Woolhouse also cited Riolan,^101^ Guillemeau, the translation of Guillemeau, and Nuck.

Although glaucoma was classically defined as being incurable, Woolhouse had to modify this teaching, given that he defined glaucoma broadly to include not only the hard eye due to phacomorphic glaucoma but also actually any disorder of the crystalline lens. Woolhouse stated that the glaucoma was amenable to the “palliative cure” of depression (couching).^43,48,74,75,101^ Of course, given the functional difficulties of aphakia, and the possibility of optic neuropathy in the acute glaucoma cases, palliation is probably a reasonable description.

The English oculist John Taylor (1703-1772) seems to have accepted Woolhouse’s teachings on what would be today called phacomorphic glaucoma. Taylor has also been accused of charlatanism but, by the standards of his day, appears to have been quite knowledgeable.^102^ In 1736, Taylor wrote of glaucoma^94^:

. . . the Volume of the Chrystalline is so greatly augmented, as to raise the Circumference of the Pupil towards the Cornea, and violently press on the Uvea.

And by this great Increase of the Volume of the Chrystalline, the Plenitude of the Globe is so greatly augmented, as to occasion Degrees of a preternatural Pressure on the immediate Organ of Sight.

And this preternatural Pressure on the Uvea and immediate Organ of Sight, is attended with Degrees of a violent Pain immediately in the Fund of the Globe . . .

Woolhouse’s student Platner has traditionally been credited^103,104^ with first calling the palpably hard eye *glaucoma* in his 1000-page-long *Institutiones Chirurgiae*, published the first of many times in 1745. According to Platner, in glaucoma,^104^

The main pathology lies in the crystalline lens which swells up. This can be recognized with the index fingers. The hard eye will resist finger pressure. In severe cases there will be pain. The color in the eye will change to sea blue. In older cases the pupil will dilate and this is called mydriasis. With that all faculty of vision disappears and amaurosis begins.

What credit should Platner receive? Platner popularized Woolhouse’s teaching that a thick crystalline lens led to a palpably hard eye, and that this condition should be called *glaucoma*. Platner’s surgical text was the standard for the period and went through multiple editions. Woolhouse’s other prominent students did not transmit this idea. Burkard David Mauchart did not write much about glaucoma. To Duddell, glaucoma implied merely a gray opacity, which could be located in the vitreous, the anterior capsule (arachnoides), or the crystalline lens.

## Softness of the Eye

Woolhouse also made a contribution with respect to the palpably soft eye. Celsus knew that after purulent exudation or trauma, an eye might be small (book 6, chapter 6)^98^: “It happens too that the eyeballs, either both or one, become smaller than naturally they ought to be. An acrid discharge of rheum in the course of ophthalmia causes this, also continuous weeping, and an injury improperly treated.” Later, Celsus noted the poor prognostic significance (book 7, chapter 7)^98^: “Neither a small nor a sunken eye is satisfactory for treatment.”

The Greek authors, such as Galen, used the term *atrophia ophthalmou* to refer to the visibly small eye.^98^ By the time of the English translation of Guillemeau, the “lean, withered, or diminished eie” was referred to as *atrophia ophthalmou, imminutio profunditas*, or *macies oculi*.^100^ As Riviere wrote of couching,

The Operation of the Needle, is more succesful, in a ful Eye; and that which keeps its natural greatness: But if the Eye be smal and decayed, it is less succesful.^99^

Today, we recognize that before a prephthisical eye becomes visibly smaller, it becomes softer (hypotonous). Woolhouse made numerous mentions that the palpably soft eye is unlikely to be curable due to what he thought was vitreous degeneration:

very frequently ye glassy humor itself is totally liquified, by ye entire solution of ye continuity of its little cellules, which one easily perceives by a touch of ye finger and, ye eye being soft and flabby. And whenever this one symptom appears or is perceived in any eye ye operation is always unfructuous as to restoring ye sight, and even ye palliative cure cannot be performed, ye globe of ye eye being full of nothing but water, nor any fibrous parts remaining, nor ye tunicks subsisting entire.^35^

likewise destroy ye fine contexture of ye cells and nervous vesicles that contain ye clear liquor that constitutes ye glassy humor and soon make an entire decomposition, or solution of ye humor known by its softness when ye eye is touched, and so ye sight must be irreparably lost.^35^

The published version of the lectures also noted that softness to palpation was a poor prognostic indicator.^43^

His student Duddell also propagated this teaching in numerous instances.^44,84^ For instance, he wrote in 1729,

These are not curable, because there is a sort of Dissolution in part of the Vitreous Humour, or its Texture is become softish, (which you may find by putting your Thumb on the upper Eye-lid; the softer you find the Globe of the Eye, the greater the Dissolution is: There is but little hope for Success where the Vitreous Humour is defective.^44^

Finally, Platner is generally credited to have first noted the poor prognosis of the palpably soft eye.^103,104^ He reported that there was a second type of glaucoma involving what he described as vitreous degeneration, secondary lens opacification, and softening of the eye.^104^

Of course, today, this condition is known as *phthisis bulbi*. “Phthisis” is from the Greek for wasting or consumption. It most commonly has been used to refer to pulmonary consumption, from tuberculosis. When used ophthalmologically in antiquity, the term phthisis referred just to the pupil (ie, miosis, or, according to Guillemeau, “a diminishing of the apple of the eie”).^100^ Phthisis of the entire eye (*phthisis oculi*) was described by the end of the 18th century.^105^ The moniker *phthisis bulbi* was used by 1811^106^ and in English in 1821.^107^

How could previous ophthalmic historians have missed the importance of Woolhouse’s observations regarding palpation of the eye? Hirschberg wrote, “It is true that Platner was a pupil of Woolhouse, but neither Woolhouse nor Saint-Yves mention palpable hardening of the eyeball in glaucoma.”^104^ Hirschberg reviewed Woolhouse’s 1717 text, which included the 1707 letter. Hirschberg might simply have missed the statement on using the finger to determine the hardness of the crystalline, or he might have interpreted it differently. Hirschberg did not have the 1721 lecture notes, and he noted the existence of the 1745 publication of Woolhouse’s lectures, but did not review them in detail. James did review the 1721 notes and made note of Woolhouse describing the palpably hard and soft eye.^33^ However, James was not reviewing the overall history of glaucoma and phthisis bulbi, and therefore, his attention may not have been drawn to Woolhouse’s priority.

## Acceptance That Couching Displaces the Lens by 1716

The major criticism of Woolhouse (among his contemporaries and among modern historians) has been that he was wrong in the debate over the nature of cataract. If one leaves aside semantics, the central point made in the new theory of Brisseau and Maitre Jan was that couching involves displacement of the opacified crystalline lens. In fact, after 1715, Woolhouse acknowledged that the new theory was correct, although he used semantic distinctions to avoid admitting defeat. He insisted that any disorder of the crystalline lens be called a glaucoma, rather than a cataract. He stated repeatedly after 1715, in both French and English, that there are 15 or 20 glaucomas of the crystalline for every real membranous cataract:

for there are certainly 20 glaucomas for one true cataract.^35^

there are certainly twenty glaucomas for one real cataract.^43^

Je leur dis même que le Glaucome étoit la maladie la plus ordinaire, puisque pour une Cataracte veritable, on rencontre jusqu’a quinze & vingt Glaucomes.^48^ [I tell them also that glaucoma is the most common malady, as, for one true cataract, one encounters up to 15 and 20 glaucomas.]

Even a brief summary of Woolhouse’s views in a letter from his student (published by Woolhouse) noted,

The only thing and the most that can be prov’d from thence is the Existence of the *Glaucoma*, which is allow’d to be an Opacity of the *Chrystalline*, and that there are indeed more of those *Glaucomata* than true Cataracts, as you have evidently prov’d it before any other Person, when you reform’d the Doctrine of the Ancients on this important Article . . .^108^

Of course, it was Brisseau, not Woolhouse, who “reformed the Doctrine of the Ancients” on the preponderance of opacities of the crystalline. This belief was a late realization on Woolhouse’s part and was not made in his initial writings after the new theory of cataract.^70–76^ Hirschberg was “puzzled” by this statement,^34^ but the most likely explanation is that Woolhouse saw the truth of the new theory but did not want to admit that he was wrong. Advocating the frequent couching of the crystalline lens is hardly the position of the classical oculist who believed the lens to be the seat of vision. The debate had degenerated into one about semantics. Woolhouse insisted that what was being couched should be called a “glaucoma” not a “cataract.”

Hirschberg devotes several pages to allegations of unprofessional behavior and even writes of Woolhouse: “He had the brains of a scholar, the hand of a gifted ophthalmic surgeon, but otherwise he is one of the worst charlatans who has ever seen the light of the day.”^34^ Why does Hirschberg call him a charlatan? Woolhouse promised manuscripts which never appeared. Hirschberg praises the advantages of Woolhouse’s conjunctival brushes but criticizes him for charging students for the brushes without revealing the method of manufacture. Woolhouse taught his students secrets he would not divulge in manuscripts. One of his manuscripts leaves out specifics and is therefore more an advertisement than a scholarly treatise. He refused to acknowledge the accomplishments of competitors. He bitterly and acrimoniously argued with his peers.^34^

But we must judge Woolhouse’s clinical acumen by the standards of his day, which included treatments, such as bloodletting, which were not efficacious and potentially harmful. Even Hirschberg admitted that Woolhouse was a scholar, well versed in both the classical and recent literature. Nowhere did Hirschberg accuse Woolhouse of providing treatments less effective than those of his contemporaries or which he (Woolhouse) believed to be ineffective.

## Other Teachings

Woolhouse was known for several advances not directly related to glaucoma. As we have reported, he was a pioneer with respect to congenital cataract surgery.^1^ As early as 1698, he wrote that he had “cured those that have been born blind.”^39^ He had performed 36 documented congenital cataract surgeries by 1721, the youngest in a patient 18 months of age.^35^ In several other areas, Woolhouse was a pioneer, although some of his contributions have been misunderstood.

## Synechiolysis

According to some accounts, Woolhouse “originated the operation of iridectomy to restore sight in cases of occluded pupil.”^109^ Others write that Woolhouse proposed iridotomy as a theoretical procedure which was then performed by the English surgeon William Cheselden.^34^ Some sources add that Cheselden had studied under Woolhouse.

In fact, there is no evidence that Woolhouse taught Cheselden. Moreover, Woolhouse actually performed operations for reconstruction of damaged pupils. Woolhouse’s texts regarding reformation of the pupil relate to repositioning of iris and pupilloplasty after uveal prolapse.^74^ His operation of “fenestration” is similar to the opening of pupillary membranes and posterior synechiolysis.^33,43^ Woolhouse teaches about cases he has actually performed and in some cases names patients.^74^ The techniques are not always described with great specificity, but we do not believe that Woolhouse recommended or performed iridotomy. Woolhouse lectured,

ye fenestration or boring a hole in ye cataract is never practiced but when ye cataract is closely adherent to ye inward borders of ye pupil, hindering its alternative opening and shutting and is by no means to be separated. Then ye oculist, must with his needle pierce ye cataract just in ye middle of ye apple of ye eye, and having made as it were but ye prick of his needle, he must, little by little, prick as it were through a parchment, as many holes as he can well make, close as it were one to another and in ye best rank and order he can lineally. Then placing ye point of his needle in ye uppermost, draw it gently downwards and so consecutively ye second and third rank by this means, he will open a good large hole in ye cataract, by which ye patient will see moderately well all manner of objects.^35^

Another type of fenestration was also described^35,43^:

Another species of Cataract we have mentioned, is woven like a web in the very hole, eye-ball, or pupil. This must be relieved first, by rubbing on the outside of the eye, to make the pupil dilate itself as much as possible; and then, having placed the patient in a moderate light, the window on the cataracted side, the oculist must cut very delicately the extremities of those fine threads, till he loosens them, and as it were unfetters the eye.^43^

In Duddell’s description of the removal of pupillary membranes, which he called “the operation of the synizizes,”^44^ he avoided cutting iris tissue. Duddell granted priority to Cheselden for his 1728 construction of the “artificial pupil” (iridotomy).^84^

Pupilloplasty and synechiolysis were important because previously, few intraocular procedures had ever enjoyed prominence—just couching and paracentesis. Even though Woolhouse popularized synechiolysis, it would be hard to grant him priority for the operation. Woolhouse knew of Richard Banister’s 1622 treatise, in which Banister wrote,

. . . after the entrance of my needle, comming to the top of the Cataract to bring it downe, I found it in the Uvea, round about the sides, with many small threds, or rather haires, which could hardly be devided, or parted asunder, because there were so many of them; yet in the end couched reasonable well . . .^110^

Woolhouse might be the first, however, to introduce the term synechiae in ophthalmology. Prior to Woolhouse, ophthalmic use of the term was not identified by searching multiple relevant databases.^89,111^ The Oxford English Dictionary notes the postclassical origin of the term and lists Woolhouse as the first to use the term in English in his 1745 lecture notes: “By the operation of the synechia, where the little ulcer, no bigger than a pin’s point, joins both the iris and the cornea together.”^112^ Moreover, the handwritten lecture notes of 1721 indicate that “. . . by ye Operation of ye Synecheie, where ye little ulcer no bigger thn a pins point, joins or tacks both ye Cornea and Iris together.”^35(p37)^ Woolhouse’s student Mauchart later published a treatise on synechiae in 1748.

## Dacryocystectomy

Woolhouse’s method of treating dacryocystitis involved extirpation of the lacrimal gland, cautery of the ethmoid bone, and placement of a golden tubule leading into the lacrimal fossa. Placement of the tubule had been done previously by Heister.^34^ Hirschberg noted Duddell’s account that the ancients were not intending to remove the lacrimal sac, and their cauterization therefore accomplished this inconsistently or incompletely.^34^ In fact, we found this statement in the manuscript of Woolhouse’s 1721 lectures.^35^ Hirschberg credits Woolhouse with being the first author to recommend dacryocystectomy, a treatment which was standard for recalcitrant dacryocystitis until the invention of dacryocystorhinostomy in 1904, and which can still be used when the latter procedure is inappropriate.^113^ Historians have known of Woolhouse’s method only because it was described by Platner and by Duddell. Woolhouse’s words on the matter have never been published. We found the following passage in the 1721 lecture manuscript^35^:

There is a particular case in ye hernia of ye lacrimal sac become varicose, and mightily distended and hard, drawing after it ye entire nasal conduit outwards, which must be extirpated entirely all at once, and cauterized likewise, which to prevent a great effusion of blood, and which to hinder ye distortion of ye eyelids which a great suppuration would infallibly produce.

## Return to England

Woolhouse’s letters place him in Paris through July 1730,^33^ after which time he returned to England. If he practiced as an oculist on his return, he did not leave any records that we could identify. Woolhouse died on January 26, 1733 (1734 by the New Style calendar).^33^

## Stephen de Beaumont

In England, his nephew Stephen de Beaumont, MD (d. 1748)^114^ continued the family tradition as an oculist. Beaumont’s biography has never been written. Beaumont was a native of France and was in Provence in about 1718. The first indication that he performed some medical procedures comes from a letter by Woolhouse in 1728: “My son Beaumont does the operation well & I’ve taken several ounces of this remedy, & do believe it sav’d myself in a great fluxion I had on my breast.”^33^ In 1729, Beaumont’s wife delivered her first child, but both mother and child died, the former after a period of illness.^33^ Woolhouse’s obituary in January 1734 noted he “Last week died at his nephew’s Mr. Beaumont, in St. Martin’s-lane.”^115^ Beaumont was executor of his estate and inherited his property.^33^ Beaumont had begun practicing as an oculist by 1736^116,117^: “Mr. Beaumont, a celebrated Occulist, couch’d in the French Hospital several Pensioners of that House, who all recover’d their Sight soon after, tho’ some of them were upwards of seventy.”^116^

Beaumont was strongly suspected of being a Jacobite sympathizer. In 1738, he was charged with speaking treasonous words—specifically drinking to the health of the Pretender (James III).^118,119^ In 1739, came the dramatic news^120,121^:

Dr. Beaumont, an eminent French Oculist in St. Martin’s-lane, was taken into Custody of his Majesty’s Messengers . . . it’s given out that the former is charg’d with aiding and assisting Mr. George Kelly in his Escape from the Tower, and for corresponding with him since at Avignon.—*Heaven defend us from a Plot*.^120^

Kelly (d. 1750), an Irish clergyman, had been imprisoned in the Tower of London after his arrest in 1722 for a pro-Jacobite conspiracy but had escaped to France in 1736. Beaumont was released on bail.

We do not know what happened with either charge, but Beaumont appears to have found social outlets in which he was accepted. He became a leader in the Masons by 1738.^114,122^ As freemasonry was essentially a British export, Beaumont permitted the establishment of a lodge in Frankfurt.^123^ In 1742, he proposed that the portrait of Frederick, Prince of Wales, be hung in the London masonic lodge.^124^ Beaumont was repaid by being named the oculist to the Prince of Wales.^125–127^ It might seem odd that the prince would include in his court an oculist ostensibly devoted to the overthrow of his father, King George II. However, the prince and his father were estranged, and the prince established an opposition court.

Beaumont died in 1748.^127^ In 1751, his widow advertised “her late Husband’s most excellent Collyrium, or Eye-Water, for curing Inflammations in the Eyes, and strengthening Weak Sight.”^128^ Thus ends the known record of the 5 generations of oculists in the Stepkins and Woolhouse family.

## Conclusions

John Thomas Woolhouse was an eye surgeon in a family of 5 generations of English oculists. He was an early adopter of paracentesis for hydrophthalmia, a condition of excess ocular tension. In response to the new theory that a cataract was an opacity of the crystalline lens, he focused attention on the term *glaucoma*, which had been applied to disorders of the lens by the ancients. He observed that swelling of the lens could lead to palpable hardness of the eye, which, due to its origin with the lens, he termed *glaucoma*. It is partly because of Woolhouse that *glaucoma*, which initially suggested a lens disorder, has come to describe an optic neuropathy for which elevated intraocular pressure is a risk factor. Woolhouse also appreciated that the soft eye was unlikely to recover vision. Woolhouse was also a pioneer with respect to surgery for synechiolysis, dacryocystectomy, and congenital cataracts.

## References

[1] Leffler CT Schwartz SG Davenport B Congenital cataract surgery during the early Enlightenment period and the Stepkins oculists. JAMA Ophthalmol. 2014;132:883–884. 24810006 10.1001/jamaophthalmol.2014.519

[2] Leffler CT Hadi TM Udupa A Schwartz SG Schwartz D A medieval fallacy: the crystalline lens in the center of the eye. Clin Ophthalmol. 2016;10:649–662. 27114699 10.2147/OPTH.S100708 PMC4833360

[3] Leffler CT Schwartz SG Giliberti FM Young MT Bermudez D What was glaucoma called before the 20th century? Ophthalmol Eye Dis. 2015;7:21–33. 26483611 10.4137/OED.S32004 PMC4601337

[4] Leffler CT Pierson K The origin of the term glaucoma: owls or light-colored eyes? J Glaucoma. 2017;26:e197–e198. 27367131 10.1097/IJG.0000000000000467

[5] Parish Register. Halstead Essex. StAndrew’s church. 12 23, 1664 John, Son of Thomas Wolhouse and Mary. https://www.freereg.org.uk/search_records/58181f57e93790ec8bc3e360?search_id=592b581533045bbfb5b1bae4&;ucf=false.

[6] Marriage of Judeth Attwood and John Stepkin. 27 8 1625 Wolverley. Ancestry.com. England, Select Marriages, 1538–1973 [database on-line]. Provo, UT: Ancestry.com Operations, Inc.; 2014 (Original data: *England, Marriages, 1538–1973* Salt Lake City, Utah: FamilySearch, 2013). http://search.ancestry.com/cgi-bin/sse.dll?_phsrc=atl112&_phstart=successSource&usePUBJs=true&gss=angs-c&new=1&rank=1&gsfn=judith&gsfn_x=NP_NN&gsln=atwood&gsln_x=NP_NN&msgdy=1625&cpxt=1&cp=4&MSAV=1&MSV=0&uidh=mf7&pcat=BMD_MARRIAGE&h=7032124&dbid=9852&indiv=1&ml_rpos=14.

[7] Robinson J The Attwood Family, With Historic Notes & Pedigrees. Sunderland, UK: Hills & Company; 1903:107 https://books.google.com/books?id=vzhLAQAAMAAJ&pg=PA107&lpg=PA107&dq=ATWOOD+worcestershire+stepkin+family+robinson&source=bl&ots=JlQ-G7B8T3&sig=mFAXwvQi5qtVjht4yEpPr6E3dSI&hl=en&sa=X&ved=0ahUKEwjt9ePjkpPUAhUGWSYKHbuRBTAQ6AEIKzAB#v=snippet&q=stepkin&f=false.

[8] Rutland C The Manuscripts of His Grace the Duke of Rutland, K.G., Preserved at Belvoir Castle. Vol 2 London, England: Her Majesty’s Stationery Office; 1889:5–347. https://archive.org/stream/manuscriptshisg00unkngoog#page/n16/mode/2up.

[9] Boswell J Johnson S The Life of Samuel Johnson, LL.D. London, England: Bell & Daldy; 1868:318.

[10] Moore N Moundeford, Thomas (1550–1630), rev. Patrick Wallis, Oxford Dictionary of National Biography, Oxford University Press, 2004 http://www.oxforddnb.com.proxy.library.vcu.edu/view/article/19435. Accessed May 29, 2017.

[11] Jenkins E Six Criminal Women. New York, NY: Duell, Sloan and Pearce; 1949:57–80.

[12] Pelling M White F BOWNE, Robert, Physicians and Irregular MedicalPractitioners in London 1550-1640 Database, London, England, 2004, British History Online. http://www.british-history.ac.uk/no-series/london-physicians/1550-1640/bowne-robert. Accessed May 29, 2017.

[13] Bramston J The Autobiography of Sir John Bramston. London, England: Camden Society; 1845:17–19.

[14] ‘Ammār ibn ‘Alī Mawṣilī, Meyerhof M. Las Operaciones de catarata de ‘Ammar ibn ‘Ali al-Mawsili. Barcelona, Spain: Laboratories del Norte de Espana; 1937:33–57.

[15] Boyle R Some Considerations Touching the Usefulness of Experimental Natural Philosophy Propos’d in Familiar Discourses to a Friend, by Way of Invitation to the Study of It. Oxford, UK: Hen. Hall for Ric, Davis, 1663:3–75.

[16] Stepkins J Will of John Stepkin or Stepkins of Wapping, Middlesex. National Archives of the UK; 1651 http://discovery.nationalarchives.gov.uk/SearchUI/Details?uri=D797587. Accessed May 29, 2017.

[17] Ivie T Alimony arraign’d, or The remonstrance and humble appeal of Thomas Ivie Esq;: from the high court of chancery, to His Highnes the Lord Protector of the Commonwealth of England, Scotland, and Ireland, &c. VVherein are set forth the unheard-of practices and villanies of lewd and defamed Women, in order to separate man and wife. London, England; 1654 https://quod.lib.umich.edu/e/eebo/A87232.0001.001/1:2?rgn=div1;view=fulltext.

[18] London, England, Church of England Baptisms, Marriages and Burials, 1538-1812 for John Stepkins. Tower Hamlets St Mary, Whitechapel 1644-1664. https://www.ancestry.com/interactive/1624/31280_194813-00365/676044?backurl=https://www.ancestry.com/family-tree/person/tree/443303/person/25778910641/facts/citation/117545962954/edit/record. Accessed May 29, 2017.

[19] Marriage of Fran Stepkin and George Williamson. Faculty Office Marriage Licenses, England, Boyd’s Marriage Indexes, 1538-1850. http://search.findmypast.com/record?id=gbprs%2fm%2f710629623%2f1.

[20] Pierce R Bath Memoirs: Or, Observations in Three and Forty Years Practice, at the Bath What Cures Have Been There Wrought, (Both by Bathing and Drinking These Waters by God’s Blessing, on the Directions of Robert Peirce, Dr. in Physick, and Fellow of the College of Physicians in London, a Constant Inhabitant in Bath, from the Year 1653. to This Present Year 1697. Bristol, UK: Hammond; 1697:169–170.

[21] Neale T Ivie T Jeffreys G The Lady Ivie’s Trial for Great Part of Shadwell . . . Before Lord Chief Justice Jeffreys in 1684 (edited by JC Fox). Oxford, UK: Clarendon Press; 1929:1–100.

[22] Sad and Lamentable News From Mapping Giving a True and Just Account of a Most Horrible and Dreadful Fire, Which Happened on Sunday the 19th. of Nov. 1682 . . . Also, Giving You a Particular Account of the Great Losses of Several Men, Namely, Sir William Warren, the Lady Ivy. London: Clarke; 1682:4.

[23] A More Full and Exact Account of That Most Dreadful Fire Which Happened at Mapping on Sunday Night the Nineteenth of This Instant November. London: D. Mallet; 1682:2.

[24] Mossam E Ivy T The famous tryal in B.R. between Thomas Neale, Esq. and the late Lady Theadosia Ivy : the 4th of June, 1684 1696:1–24.

[25] Marriage of Ann Stepkin and John Woolhouse. St. Olave Silver Street. Boyd’s Marriage Indexes, 1538-1850. 1627 http://search.findmypast.com/record?id=gbprs%2fm%2f754625038%2f2.

[26] Venn J Venn JA Alumni Cantabrigienses; A Biographical List of All Known Students, Graduates and Holders of Office at the University of Cambridge, from the Earliest Times to 1900. Part 1. Vol 4 London, England: Cambridge University Press; 1927:462 https://archive.org/stream/p1alumnicantabri04univuoft#page/462/mode/2up.

[27] Blackburne DFH Thomas Woollhouse to Williamson. Calendar of State Papers, Domestic Series, March 1st, 1675, to February 29th, 1676. London, England: Mackie and Co; 1907:4 https://archive.org/stream/calendarstatepa00levagoog/calendarstatepa00levagoog_djvu.txt.

[28] Acland CL Round JH Register of the Scholars Admitted to Colchester School, 1637-1740. Colchester, UK: Wiles & Son; 1897:40 https://archive.org/stream/registerscholar00schogoog/registerscholar00schogoog_djvu.txt.

[29] “Woolhouse, Thomas. Page of the presence . . . reappointed James 1st . . . dead by 1688” The Royal Archives. Royal Household Staff 1526-1924. Volume: Royal Household Index 1660–1901. http://search.findmypast.co.uk/record/browse?id=gbor%2fhrh%2fhousehold%2fgb_w-z%2f00468.

[30] Woolhouse T Will of Thomas Woolhouse, Page of the Present Chamber to His Majesty of Saint Margaret Westminster, Middlesex, 6 1688 National Archives of the UK. http://discovery.nationalarchives.gov.uk/details/r/D729903. Accessed July 16, 2013.

[31] Woolhouse JT Thomas Woolhouse, Esq . . . Related to the Late Famous Oculist, My Lady Ivy Post Man and the Historical Account. 3 27-29, 1701: Issue 817:3.

[32] Lee S, ed. Dictionary of National Biography. Vol 28 New York, NY: MacMillan and Co; 1891:33 http://books.google.com/books?id=SEZegCvIjb0C&q=henry+howard#v=snippet&q=henry%20howard&f=false.

[33] Burial of Thomas Woolhouse. 5 30, 1688 St. Margaret. Westminster Burials Transcription. http://search.findmypast.com/record?id=gbprs%2fd%2f490899632%2f1.

[34] James RR Woolhouse (1666–1733-4). Br J Ophthalmol. 1934;18:193–217. 18169190 10.1136/bjo.18.4.193 PMC511666

[35] Hirschberg J Blodi FC The History of Ophthalmology. Vol 3 The Renaissance of Ophthalmology in the Eighteenth Century (Part One) Bonn, Germany: J. P. Wayenborgh Verlag; 1984:5–369.

[36] Woolhouse JT A Treatise of ye Cataract & Glaucoma. London, England: Royal Society of Medicine Manuscript; 1721:4–66.

[37] Woolhouse J Page of the presence to Charles II. April 26, 1681. The Royal Archives. Royal Household Staff-1526-1924. Volume: Royal Household Index 1660–1901. http://search.findmypast.co.uk/record?id=gbor%2fhrh%2fhousehold%2fgb_w-z%2f00465&parentid=gbprs%2fhrh%2f88011465%2f1.

[38] Woolhouse JT Le Roy d’Angleterre, scachant qu’il y a des personnes a Paris qui se dissent Oculistes. Mercure Galant. Paris. Chez Michel Brunet. 10 1707:299–305. http://gallica.bnf.fr/ark:/12148/bpt6k6310148c/f303.image.r=woolhouse.

[39] Calendar of the Manuscripts of the Marquis of Bath. Vol 3 Hereford, UK: Anthony Brothers Ltd; 1908:290–295. https://books.google.com/books?id=yDfYAAAAMAAJ&pg=PA290&lpg=PA290&dq=john+woolhouse+matthew+prior+1698&source=bl&ots=jdE2PEcXsO&sig=6jnROQWZPHqCBCIkZvamPyZoRyo&hl=en&sa=X&ved=0ahUKEwiwg7CS75PUAhVG6CYKHXWgB-4Q6AEIKDAA#v=onepage&q=woolhouse&f=false.

[40] Woolhouse JT Recueil d’instrumens pour les opérations manuelles des yeux. Par Mr Woolhouse oculiste anglois, serviteur du roy de la Grande-Bretagne. Paris, France: Chez Laurent d’Houry; 1696:1–10.

[41] Calendar of the Stuart Papers belonging to His Majesty the King. Vol 1 London, England: Mackie &Co; 1902:215 http://books.google.com/books?id=5sYKAAAAYAAJ&pg=PA215&lpg=PA215&dq=%22john+thomas+woolhouse%22+letter&source=bl&ots=VSQN75_bn-&sig=y6O_61WeTc405TuBrqbOXP-_fHI&hl=en&sa=X&ei=ETC2UbbSHLSG0QGgvoDYCg&ved=0CGUQ6AEwCQ#v=onepage&q=%22john%20thomas%20woolhouse%22%20letter&f=false.

[42] Woolhouse JT Mr. Woolhouse, Gentil-homme Anglois. Mercure Galant. Paris, France: Chez Michel Brunet; 1696:53–57. http://gallica.bnf.fr/ark:/12148/bpt6k62588254/f53.image.r=woolhouse.langEN. Accessed July 16, 2013.

[43] Gerebtzoff M History of Civilization in Russia. Reviewed in: British Foreign Medical Review. London, England: John Churchill; 1862:298 http://books.google.com/books?id=ZlhYAAAAMAAJ&pg=PA298&dq=woolhouse+peter+the+great+russia+oculist&hl=en&sa=X&ei=zym2UdzJI-PN0gHXrIHYAg&ved=0CDcQ6AEwAA#v=onepage&q=woolhouse%20peter%20the%20great%20russia%20oculist&f=false.

[44] Woolhouse JT A Treatise of the Cataract and Glaucoma: In Which the Specific Distinctions of Those Two Diseases, and the Existence of Membranous Cataracts, Are Clearly Demonstrated . . . Compiled from the Dictates of the Late Learned and Ingenious Mr. Woolhouse, as Taken from Him in Writing, by One of His Pupils. London, England: M. Cooper, at the Globe in Pater-Noster-Row; and G. Woodfall, at the King’s-Arms, Charing-Cross; 1745:3–116.

[45] Duddell B A Treatise of the Diseases of the Horny-Coat of the Eye, and the Various Kinds of Cataracts. London, England: John Clark; 1729:4–230.

[46] Wyman AL Benedict Duddell: pioneer oculist of the 18th century. J Royal Society Medicine. 1992;85:412–415. PMC1293550 1629852

[47] Grzybowski A McGhee CN The early history of keratoconus prior to Nottingham’s landmark 1854 treatise on conical cornea: a review. Clin Exp Optometry. 2013;96:140–145. 10.1111/cxo.12035 23414219

[48] Hirschberg J Blodi FC The History of Ophthalmology. Vol 4 The Renaissance of Ophthalmology in the Eighteenth Century. (Part Two) Bonn, Germany: J. P. Wayenborgh Verlag; 1984:16–47.

[49] Woolhouse JT LeCerf C Dissertations scavantes et critiques de monsieur de Woolhouse sur la cataracte et le glaucoma. Offenbach sur le Main: Bonaventure de Launoy; 1717:21–299. http://www2.biusante.parisdescartes.fr/livanc/index.las?cote=30850&do=chapitre.

[50] Kovacs J Unschuld PU Essential Subtleties on the Silver Sea. The Yin-hai jing wei: A Chinese Classic on Ophthalmology. Berkeley, CA: University of California Press; 1998:4–211.

[51] Fan KW Couching for cataract and Sino-Indian medical exchange from the sixth to the twelfth century AD. Clin Exp Ophthalmol. 2005;33:188–190. 15807830 10.1111/j.1442-9071.2005.00978.x

[52] Cleyer A Specimen Medicinae Sinicae. Francofurtum. 1682:6–289. https://books.google.com/books?id=VhdAAAAAcAAJ&q=ophthalmia#v=onepage&q=radicale&f=false.

[53] Verwaal RE Hippocrates Meets the Yellow Emperor: On the Reception of Chinese and Japanese Medicine in Early Modern Europe [master’s thesis]. 2010 http://dspace.library.uu.nl/handle/1874/179050.

[54] Ten Rhyne W Wilhelmi ten Rhyne . . . Dissertatio de arthritide: Mantissa schematica De acupunctura et orationes tres, I. De chymiae ac botaniae antiquitate & dignitate II. De physiognomia III. De monstris:singula ipsius authoris notis illustratur. Londini: Impensis R. Chiswell; 1683:184–186. https://babel.hathitrust.org/cgi/pt?id=ucm.5323751734;view=1up;seq=210.

[55] Bonet T A guide to the practical physician shewing, from the most approved authors, both ancient and modern, the truest and safest way of curing all diseases, internal and external, whether by medicine, surgery, or diet. Published in Latin by the learn’d Theoph. Bonet, physician at Geneva. And now rendred into English, with an addition of many considerable cases, and excellent medicines for every disease. Collected from Dr. Waltherus his Sylva medica. by one of the Colledge of Physicians, London. London: Thomas Flesher, at his house over against Distaff Lane in the Old Change; 1686:31–393.

[56] Valentini MB Mich. Bern. Valentini Historia moxae: cum adjunctis in fine meditationibus de podagra ad . . . Andream Cleyerum . . . perscripta. Lugduni Batavorum: Van der Aa; 1686:6 https://archive.org/stream/bub_gb_C_EbM2DpcT8C#page/n5/mode/2up.

[57] Valentini MB Museum Museorum, Oder Vollständige Schau-Bühne Aller Materialien und Specereÿen Nebst deren Natürlichen Beschreibung. Franckfurt am Mäyn Zunner; 1704.

[58] Valentini MB Observatio LXX. Hydrophthalmia puncturâ acus percurata. Miscellanea Curiosa Sive Ephemeridum Medico-Physicarum Germanicarum Academiae Imperialis Leopoldinae Naturae Curiosorum. 12 2 Ann 6. 1687:159–160. http://books.google.com/books?id=3X1EAAAAcAAJ&pg=PA159&lpg=PA159&dq=valentini+hydrophthalmia+6&source=bl&ots=1yVRqTY9TL&sig=RN6SqRVwUXMgAFZQYzbgo9dcE1I&hl=en&sa=X&ei=sQrKUZmSKufw0QHZ2ICoAw&ved=0CDMQ6AEwAg#v=onepage&q=hydrophthalmia&f=false.

[59] Nuck A Sialographia et Ductuum Aquosorum Anatome Nova. Lugduni Batavorum: apud Jordanum Luchtmans, 1695:120–126. http://babel.hathitrust.org/cgi/pt?id=ucm.532736105x;view=1up;seq=144.

[60] Hirschberg J Blodi FC The First Half of the Nineteenth Century. Part Four. Great Britain. Vol 8A. Bonn, Germany: Wayenborgh; 1987:61.

[61] Leffler CT Wainsztein RD The first cataract surgeons in Latin America: 1611-1830. Clin Ophthalmol. 2016;10:679–94. 27143845 10.2147/OPTH.S105825 PMC4841434

[62] Mauchart BD Sarwey TA Paracentesis oculi in hydrophthalmia et amblyopia senum. Tubingen: Sigmundino; 1744:9.

[63] Turberville D Letter from Dr. Turbervile of salisbury containing some considerable observations in the practise of physic. Phil Trans Royal Society. 1684–1685;15:839–840.

[64] Chambers E A Supplement to Mr. Chambers’s Cyclopædia: or, Universal Dictionary of Arts and Sciences. London, England: Innys, Vol 1 1753:n.p.

[65] Velpeau A Pattison GS New Elements of Operative Surgery. Washington, DC: Duff Green; 1835:372–373.

[66] Mark HH Buphthalmos: early glaucoma history. Acta Ophthalmol. 2011;89:591–594. 20529079 10.1111/j.1755-3768.2009.01783.x

[67] Middlemore R A treatise on the diseases of the eye and its appendages. London, Longman, Rees, Orme, Brown, Green, and Longman, 1835:475–483. https://archive.org/stream/treatiseondiseas02midd#page/476/mode/2up.

[68] Boerhaave H Dr. Boerhaave’s academical lectures on the theory of physic. Being a Genuine Translation of His Institutes and Explanatory Comment. London, England: J. Rivington, 1757: Vol 4:99.

[69] Woolhouse JT De la maladie des yeux. Mercure Galant. Paris, France: Michel Brunet; 12 1696:265–272. http://gallica.bnf.fr/ark:/12148/bpt6k6268932w/f265.image.r=woolhouse.langEN.

[70] Woolhouse JT Memoire, De Plusieurs de Couvertes & Operations nouvelles en Anatomie & Chirurgie faites sur les yeux, par Mr. de Woolhouse. Mercure Galant. 1703:107–142. http://gallica.bnf.fr/ark:/12148/bpt6k6278988j.r=woolhouse.langEN.

[71] Woolhouse JT Reflexions Sur le Systeme pretendu nouveau de Mre-Antoine Maitre-Jan &c. Mercure Galant. Paris, France: Brunet 10 1708:10,33,41,54. http://gallica.bnf.fr/ark:/12148/bpt6k6307576z/f12.image.r=woolhouse.langEN.

[72] Woolhouse JT Troisome suite de l’Ouvrage de Mr. de Woolhouse. Mercure Galant. 1 1709:59–103. http://gallica.bnf.fr/ark:/12148/bpt6k6293108r/f63.image.r=woolhouse.langEN.

[73] Woolhouse JT Quatrième suite de l’ouvrage de Mr. de Woolhouse. Mercure Galant. Febraury 1709:64–98. http://gallica.bnf.fr/ark:/12148/bpt6k6307606n/f66.image.

[74] Woolhouse JT Cinquième suite de l’ouvrage de Mr de Woolhouse. Mercure Galant. 3 1709:68–109. http://gallica.bnf.fr/ark:/12148/bpt6k63092665/f72.image.r=woolhouse.langEN.

[75] Woolhouse JT Expériences des différentes opérations manuelles et des guérisons spécifiques, que le Sieur de Woolhouse, gentil-homme & oculiste du Roi d’Angleterre, a toûjours pratiquées aux yeux. Paris, France: Chez Guillaume Valleyre, ruë Saint Jacques, à la Ville de Riom 1711:3–18. http://www2.biusante.parisdescartes.fr/livanc/index.las?cote=90958x143x02&do=pages.

[76] Woolhouse JT Expériences des différentes opérations manüelles et des guérisons spécifiques, que le sieur de Woolhouse, gentil-homme & oculiste anglois, a toujours pratiquées aux yeux -Paris, France: Chez Guillaume Valleyre, ruë Saint Jacques, à la Ville de Riom. 1712:19–25.

[77] Woolhouse JT Observations Critiques de M. De Woolhouse. Le Journal des sçavans. Académie des inscriptions et belles-lettres. Vol. XXII Paris 1714 Cuson. pp. 343–348. http://gallica.bnf.fr/ark:/12148/bpt6k56563q/f345.image.

[78] Woolhouse JT Avertissement pour les Maladies de l’Oeil. Le Nouveau Mercure. 5 1721:188–191. http://gallica.bnf.fr/ark:/12148/bpt6k63284255/f195.image.r=woolhouse.langEN.

[79] Mauchart BD Beger CP Hydrophthalmia Sive Hydrops Oculi. Tubingae: Bauhofius atque Franckius, 1744:11–13. https://books.google.com/books/reader?id=19k-AAAAcAAJ&printsec=frontcover&output=reader&pg=GBS.PP12.

[80] Boissier de la Croix de Sauvages F, Wallis G. Nosologia Methodica Oculorum: Or, a New Treatise on the Diseases of the Eyes. London, England: Alex. Hogg, 1785:228–234.

[81] Lawrence W A Treatise on the Diseases of the Eye. London, England: Churchill; 1833:655–656.

[82] Leffler CT Schwartz SG Hadi TM Salman A Vasuki V The early history of glaucoma: the glaucous eye (800 BC to 1050 AD). Clin Ophthalmol. 2015;9:207–215. 25673972 10.2147/OPTH.S77471 PMC4321651

[83] Le Grand A Bome R An Entire Body of Philosophy According to the Principles of the Famous Renate Des Cartes in Three Books . . . Written Originally in Latin by the Learned Anthony Le Grand. London, England: Samuel Roycroft, 1694:211.

[84] Duddell B An Appendix to the Treatise of the Horney-Coat of the Eye, and the Cataract. With an Answer to Mr. Cheselden’s Appendix, Relating to His New Operation upon the Iris of the Eye. London, England: E. Howlatt; 1733:1–182.

[85] Saint-Yves C A new treatise of the diseases of the eyes. Containing proper remedies, and describing the chirurgical operations requisite for their cures. With some new discoveries in the structure of the eye, that demonstrate the immediate organ of vision. London, 1741:261–266.

[86] Mery J Oeuvres complètes de Jean Méry. Paris. 1888:60–541. http://gallica.bnf.fr/ark:/12148/bpt6k28139m/f608.image.r=Oeuvres%20completes%20de%20Mery%20.langEN.

[87] Arrachart JN Mémoires, dissertations, et observations de chirurgie. Paris 1805:115–116. http://gallica.bnf.fr/ark:/12148/bpt6k56589739.r=babelin.langEN.swf.

[88] Leffler CT Schwartz SG Davenport B Randolph J Busscher J Hadi T Enduring Influence of Elizabethan Ophthalmic Texts of the 1580s: Bailey, Grassus, and Guillemeau. Open Ophthalmol J. 2014;8:12–18. 24959303 10.2174/1874364101408010012 PMC4066364

[89] Early English Books Online Text Creation Partnership. http://quod.lib.umich.edu/e/eebogroup/. Accessed September 10, 2016.

[90] Riolan J (le père) Ioannis Riolani ambiani medici parisiensis, viri clarissimi opera omnia Parisiis: ex officina Plantiniana, 1610:443–444. http://www2.biusante.parisdescartes.fr/livanc/?cote=00326&do=chapitre. Accessed April 29, 2013.

[91] Riolan J A Sure Guide, or, The Best and Nearest Way to Physick and Chirurgery. London, England: Peter Cole; 1657:142.

[92] Nouvelles Observations sur la cataracte proposées àl’Académie royale des sciences, le 18 novembre 1705, par Mr Brisseau le fils. Mémoires pour l’histoire des sciences & des beaux arts. 1706;8:2022–2023.

[93] Brisseau M Traité de la cataracte et du glaucoma. Paris, France: Laurent d’Houry; 1709.

[94] Taylor J A New Treatise on the Diseases of the Chrystalline Humour of a Human Eye: Or, of the Cataract and Glaucoma. London, England: James Roberts; 1736:28–29.

[95] O’Halloran. A New Treatise on the Glaucoma, or Cataract. Dublin: Powell; 1750.

[96] Heister L Laurentii Heisteri . . . De cataracta, glaucomate et amaurosi tractatio: in qua multae novae opiniones & inventa contra vulgatas medicorum, chirurgorum, philosophorum nec non mathematicorum sententias continentur. Altorfi: Literis Iod. Guil. Kohlesii; 1713.

[97] Leffler CT Schwartz SG Stackhouse R Davenport B Spetzler K Evolution and impact of eye and vision terms in written English. JAMA Ophthalmol. 2013;131:1625–1631. 24337558 10.1001/jamaophthalmol.2013.917

[98] Hirschberg J Blodi FC The History of Ophthalmology. Vol 1, Antiquity. Bonn, Germany: Wayenborgh; 1982:228–331.

[99] Rivière L Culpeper N (tr.), Cole A (tr.), Rowland W (tr.). The Practice of Physick in Seventeen Several Books Wherein Is Plainly Set Forth the Nature, Cause, Differences, and Several Sorts of Signs: Together With the Cure of All Diseases in the Body of Man. London, England: Peter Cole; 1655:70–71.

[100] Guillemeau J Hunton A Bailey W A Worthy Treatise of the Eyes Contayning the Knowledge and Cure of One Hundred and Thirteen Diseases. London, England: Printed by Robert Waldegraue for Thomas Man and William Brome; 1587: 7–196.

[101] Woolhouse JT Sixième suite de l’Ouvrage de Mr de Woolhouse. Mercure Galant. 4 1709:42–81. http://gallica.bnf.fr/ark:/12148/bpt6k6291028j/f44.image.r=woolhouse.langEN.

[102] Schwartz SG Leffler CT Grzybowski A Koch HR Bermudez D The Taylor Dynasty: three generations of 18th-19th century oculists. Historia Ophthalmologica Internationalis. 2015;1:67–81.

[103] Terson MA Les premiers observateurs de la durété de l’oeil dans le glaucome. Archives d’Ophtalmologie. 1907;625–630.

[104] Hirschberg J Blodi FC The History of Ophthalmology. Vol 6 The First Half of the Nineteenth Century (Part Two). Bonn, Germany: J. P. Wayenborgh Verlag; 1986:154–164.

[105] Horrebow M Tractatus de oculo humano ejusque morbis. Havniae, N. Christensen; 1792:61–62. http://books.google.com/books?hl=en&lr=&id=U80WAAAAQAAJ&oi=fnd&pg=PA1&dq=phthisis&ots=vsveXsRare&sig=LWQDvn2-YdNRuRzXtu5BQpiGiyE#v=onepage&q=phthisis&f=false.

[106] Scheuring J Parallele der Vortheile und Nachtheile der vorzüglichsten Operationsmethoden des grauen Staares. Bamberg. Goebhardt. 1811:39 http://books.google.com/books?hl=en&lr=&id=M-0-AAAAcAAJ&oi=fnd&pg=PA1&dq=%22phthisis+bulbi%22&ots=tb1cYjNnso&sig=AbOlymulRw4bblFMnVFrgARRnek#v=onepage&q=phthisis&f=false.

[107] Leffler CT Randolph J Stackhouse R Davenport B Spetzler K Monteath’s translation of Weller: an underappreciated trove of ophthalmology Lexicon. Arch Ophthalmol. 2012;130:1356–1357. 10.1001/archophthalmol.2012.2084 23044972

[108] The Dispute which has long subsisted among the most famous Oculists and Chirurgeons in Europe, about the Difference of the Glaucoma and Cataract, continuing still. Weekly Medley. Issue 46, 8 16, 1729:2.

[109] McConnell A Woolhouse, John Thomas (1666–1734), Oxford Dictionary of National Biography, Oxford University Press; 2004 http://www.oxforddnb.com.proxy.library.vcu.edu/view/article/29954. Accessed June 20, 2017.

[110] Banister R Guillemeau J Hunton A Weyer J Textor B Bailey W A Treatise of One Hundred and Thirteene Diseases of the Eyes, and Eye-Liddes. London, England: Felix Kyngston, for Thomas Man, dwelling in Pater-noster-row, at the sign of the Talbot, 1622.

[111] Gale Eighteenth Century Collections Online. http://www.gale.com/primary-sources/eighteenth-century-collections-online. Accessed June 20, 2017.

[112] Synechia. Oxford English Dictionary Online. 3rd ed. 2014 http://www.oed.com.proxy.library.vcu.edu/view/Entry/196463?redirectedFrom=synechiae#eid.

[113] Matayoshi S Van Baak A Cozac A Sardinha M Dias Fernandes JBV da Mota Moura E Dacryocystectomy: indications and results. Orbit. 2004;23:169–173. 15545130 10.1080/01676830490504133

[114] Anderson J The New Book of Constitutions of the Ancient and Honourable Fraternity of Free and Accepted Masons. London, England: Brothers Cæsar Ward and Richard Chandler; 1738:139.

[115] Last week died at his nephew’s Mr. Beaumont . . . John-Thomas Woolhouse. Penny London Post. Issue 67, 1 28, 1734:2.

[116] On Tuesday last Mr. Beaumont, a celebrated Occulist, couch’d in the French Hospital several Pensioners. Old Whig or The Consistent Protestant. Issue 66, 6 10, 1736:3.

[117] On Monday last Mr. Beaumont, a famous Oculist. Daily Post. 6 4, 1736: Issue 5219:3.

[118] Rex v Stephen Beaumont for drinking the Pretender’s health on 10 June, 11 Geo II (1738): Middlesex sessions. UK National Archives. 1738. Manuscript Number TS 11/424. (2 pp.).

[119] Osborne T A catalogue of thirty thousand volumes, (with the prices printed) of several libraries just purchas’d; particularly the library of William Kynaston, Esq. London. 1749:21.

[120] Dr. Beaumont, an eminent French Oculist in St. Martin’s-lane, was taken into Custody. London Evening Post. 1 6-9, 1739: Issue 1740:2.

[121] An Oculist in St. Martin’s Lane . . . was taken into Custody. Common Sense or The Englishman’s Journal. 1 13, 1739: Issue 102:3.

[122] The Pocket Companion and History of Free-Masons, Containing Their Origine, Progress, and Present State: An Abstract of Their Laws, Constitutions, Customs. London, England: J. Scott; 1754:189.

[123] Gould RF A Library of Freemasonry: Comprising Its History, Antiquities, Symbols, Constitutions, Customs, Etc. John C. Yorston, 1906:37 http://books.google.com/books?id=dmouAAAAYAAJ&pg=PA37&lpg=PA37&dq=%22brother+beaumont%22+oculist&source=bl&ots=ehCFgtLtde&sig=w1KrI57UMOAfoJmPcqM_sw5isVM&hl=en&sa=X&ei=SLBEU6qWF6izsQSjy4GIDQ&ved=0CCkQ6AEwAA#v=onepage&q=%22brother%20beaumont%22%20oculist&f=false.

[124] At a full Lodge of Free-Masons . . . Dr. Beaumont. Daily Post. 2 26, 1742: Issue 7013:3.

[125] An account of the proceedings of the governors of St. George’s hospital near Hyde-Park-Corner, from its first institution, October the nineteenth 1733. to the twenty-eighth of December 1743. St. George’s Hospital. London, England; 1744:3.

[126] An account of the proceedings of the governors of St. George’s hospital near Hyde-Park-Corner, from its first institution, October the nineteenth 1733. to the thirty-first of December 1746. St. George’s Hospital. London, England; 1747:3.

[127] A few days ago died Dr. Beaumont, Oculist to his Royal Highness the Prince of Wales. Penny London Post or The Morning Advertiser. 11 7-9, 1748: Issue 1023:2.

[128] Beaumont. The widow of the late Dr. Beaumont. General Advertiser. 8 13, 1751: Issue 5246:3.

